# Genetic Control over mtDNA and Its Relationship to Major Depressive Disorder

**DOI:** 10.1016/j.cub.2015.10.065

**Published:** 2015-12-21

**Authors:** Na Cai, Yihan Li, Simon Chang, Jieqin Liang, Chongyun Lin, Xiufei Zhang, Lu Liang, Jingchu Hu, Wharton Chan, Kenneth S. Kendler, Tomas Malinauskas, Guo-Jen Huang, Qibin Li, Richard Mott, Jonathan Flint

**Affiliations:** 1 Wellcome Trust Centre for Human Genetics, University of Oxford, Roosevelt Drive, Oxford, Oxfordshire OX3 7BN, UK; 2 Department and Graduate Institute of Biomedical Sciences, College of Medicine, Chang Gung University, Tao-Yuan 33302, Taiwan; 3 BGI-Shenzhen, Floor 9 Complex Building, Beishan Industrial Zone, Yantian District, Shenzhen, Guangdong 518083, China; 4 Virginia Institute for Psychiatric and Behavioral Genetics, Virginia Commonwealth University, Richmond, VA 23298, USA; 5 Cold Spring Harbor Laboratory, Beckman Building, One Bungtown Road, Cold Spring Harbor, NY 11724, USA

## Abstract

Control over the number of mtDNA molecules per cell appears to be tightly regulated, but the mechanisms involved are largely unknown. Reversible alterations in the amount of mtDNA occur in response to stress suggesting that control over the amount of mtDNA is involved in stress-related diseases including major depressive disorder (MDD). Using low-coverage sequence data from 10,442 Chinese women to compute the normalized numbers of reads mapping to the mitochondrial genome as a proxy for the amount of mtDNA, we identified two loci that contribute to mtDNA levels: one within the *TFAM* gene on chromosome 10 (rs11006126, p value = 8.73 × 10^−28^, variance explained = 1.90%) and one over the *CDK6* gene on chromosome 7 (rs445, p value = 6.03 × 10^−16^, variance explained = 0.50%). Both loci replicated in an independent cohort. *CDK6* is thus a new molecule involved in the control of mtDNA. We identify increased rates of heteroplasmy in women with MDD, and show from an experimental paradigm using mice that the increase is likely due to stress. Furthermore, at least one heteroplasmic variant is significantly associated with changes in the amount of mtDNA (position 513, p value = 3.27 × 10^−9^, variance explained = 0.48%) suggesting site-specific heteroplasmy as a possible link between stress and increase in amount of mtDNA. These findings indicate the involvement of mitochondrial genome copy number and sequence in an organism’s response to stress.

## Highlights

• Loci near TFAM and CDK6 contribute to variation in the amount of mtDNA
• Mutations accumulate in the mtDNA of cases of major depression
• Animal experiments show that heteroplasmy can be induced by chronic stress
• Amount of mtDNA is associated with site-specific heteroplasmy

## Teaser

Having previously shown that cases of major depressive disorder have increased mtDNA, that exposure to stress is likely responsible, and that the changes in amount of mtDNA are reversible, Cai et al. investigate in this paper the genetic control and consequences of this change and how this relates to major depressive disorder.

## Introduction

The number of mtDNA molecules appears to be tightly regulated, as inferred from the constant amount of mtDNA per mitochondrion in different cells in the presence of marked variation in the number of mitochondria between cell types [1]. The mechanisms involved are largely unknown [2, 3]. One of the components of the mtDNA replication machinery, mitochondrial transcription factor A (*TFAM*), appears to be a key transcription factor controlling the amount of mtDNA [4], but it is still not known how that signal is used by cells to count the amount of mtDNA that they require. The observation that the number of mitochondria predicts cell division better than other measures such as cell volume or cell size [5] suggests replication of mtDNA and mitochondrial biogenesis, while autonomous, may be regulated by cell-cycle machinery and vice versa.

We recently observed that the amount of mtDNA alters in response to external stress: there was significantly more mtDNA in the saliva and blood of people with major depression than in controls [6]. Chronic stress also altered the amount of mtDNA in mouse tissues, and, at least in part, was restored to pre-stress levels following cessation of stress [6]. Changes in cellular composition could not account for these observations [6], suggesting that the mtDNA alterations reflected changes within cells, and corticosteroid signaling down the hypothalamic-pituitary axis may be involved in causing these changes since injection of corticosterone alone can recapitulate effects of chronic stress.

These findings raised questions as to what contributes to changes in the amount of mtDNA, how that is related to major depressive disorder, and what are the consequences for a cell of maintaining a high turnover of mtDNA molecules, which have relatively high mutation rates. We set out to use genotypes and measures of mtDNA levels from whole-genome low-coverage sequencing on 10,442 Han Chinese women recruited in the CONVERGE (China, Oxford, and Virginia Commonwealth University Experimental Research on Genetic Epidemiology) Consortium of Major Depressive Disorder (MDD). Using this dataset, we set out to perform the first genome-wide association study (GWAS) on mtDNA levels to discover what might be involved in the molecular pathways controlling mtDNA levels. We further ask whether variation in mtDNA sequences contributes to variations in the amount of mtDNA and whether such variation is related to MDD.

## Results

**Figure 1:**
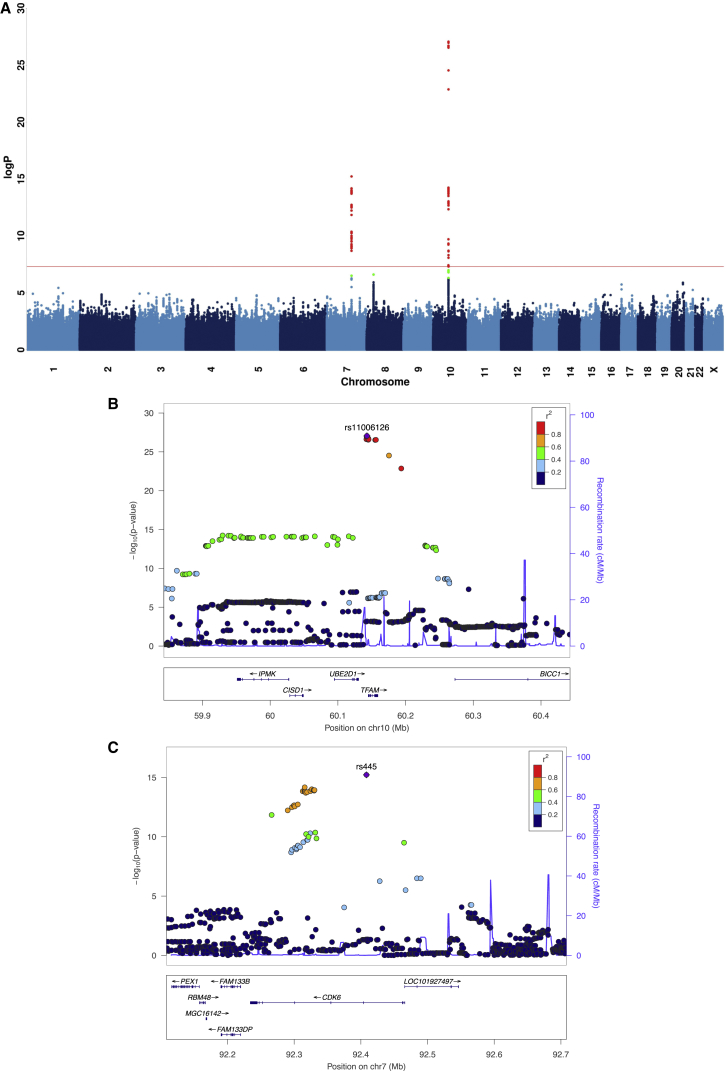
Two Loci Associated with mtDNA Manhattan plot of genome-wide association for amount of mtDNA (A). Detailed views of two loci associated with amount of mtDNA over the *TFAM* region on chromosome 10 at 60.1 Mb (B) and the *CDK6* gene on chromosome 7 at position 92.4 Mb (C) are shown. The −log10 p values of imputed SNPs associated with amount of mtDNA are shown on the left y axis. The horizontal axis gives chromosomal position in megabases (Mb). Genes within the regions are shown in the bottom panels. Linkage disequilibrium of each SNP with top SNP, shown in large purple diamond, is indicated by its color. The plots were drawn using LocusZoom [9]. See also Figure S1.

CONVERGE obtained sequence from Chinese women with a mean coverage of 1.7× of the nuclear genome [7]. For this study, we used sequences from 5,224 women with MDD and 5,218 controls, and imputed allele dosages at 6,242,619 SNPs across the nuclear genome (details on imputation methodology and genotype quality are given in [7]). We began by determining that the amount of mtDNA, defined as the number of reads mapping to the mitochondrial genome over read coverage of the nuclear genome, controlled for age of subject and sequencing batch (Experimental Procedures), is a heritable trait, with an estimated SNP-based heritability [8] of 15.6% (SE = 5.1%; p value = 1.06 × 10^−3^). We then mapped variation in the amount of mtDNA as a quantitative trait and identified two loci that exceeded genome-wide significance (genomic control lambda [λ] = 1.017; Figure 1A). One locus is within the *TFAM* gene on chromosome 10 (top SNP rs11006126, p value = 8.73 × 10^−28^, variance explained by gene = 1.90%, Figure 1B), and the second lies over the *CDK6* gene on chromosome 7 (top SNP rs445, p value = 6.03 × 10^−16^, variance explained by gene = 0.50%, Figure 1C).

The most highly associated SNP (rs11006126) resides in the 3′ region of the gene *TFAM*. The most highly associated SNPs at the *CDK6* locus (rs445) lie in intron 1 of the *CDK6* gene. Both associations replicated in a cohort of 1,753 samples from the Avon Longitudinal Study of Parent and Children (ALSPAC) [10], that forms part of the UK10K study [11]. Notably DNA from this cohort was obtained from blood. Association results for the top two SNPs (rs445 and rs11006126) in separate and joint analyses are summarized in Table 1. All SNPs associated with the amount of mtDNA in the CONVERGE study at p values <10^−6^ are shown in Table S1. Our measure of the amount of mtDNA from sequencing data is highly correlated with that from another recently published method [12]. GWAS using measures from both methods of quantification gave highly similar results (data not shown).

We next asked whether the mtDNA sequence itself governs the alterations in mtDNA quantity, and whether cycles of replication might result in mutations in the mtDNA molecule. The latter was considered likely given the relatively higher mutation rate of mtDNA compared to genomic DNA. We therefore set out to identify variant sites in the 16 Kb mitochondrial genome and to quantify their frequency both between and within individuals (coverage of each individual’s mitochondrial genome is approximately 100-fold).

**Figure 2:**
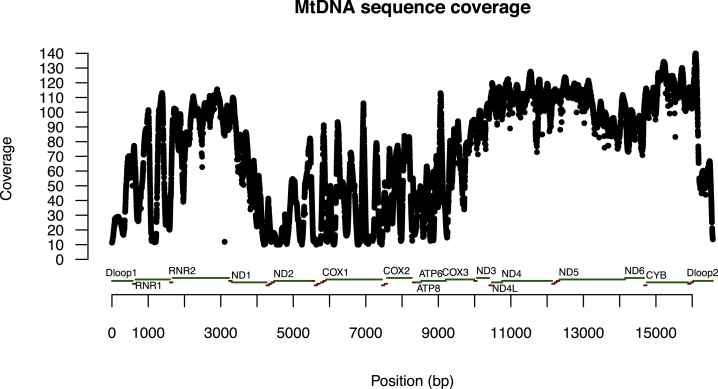
Accessibility of mtDNA for Variant Calling Mean per site read depth across 10,442 samples for all sites on the mtDNA. Reads included in the per site read count have passed the following quality control criteria: (1) mapping quality >59, (2) both ends of paired-end read map uniquely to the mtDNA reference NC_012920.1, (3) they do not contain mismatches to the mtDNA reference with total length of 5 bp or above, and (4) base at site in question has base quality of higher than 20. Sites with more than 10% samples with read depth less than 10 would not be used for calling homoplasmic variants, and sites with more than 10% samples with read depth less than 50 would not be used for calling heteroplasmic variants. See also Experimental Procedures.

Analysis of variants in mtDNA is subject to a number of potential confounds, which we took care to avoid. First, we had to ensure that the sequence variants we obtained were truly in the mitochondrial and not in the nuclear genome (since the latter contains partial copies of the former [13]), and also that they were not derived from exogenous sources (DNA for this project was extracted from saliva). After applying stringent quality-control filters and criteria (Experimental Procedures), 89% of the mitochondrial genome was considered sufficiently unique for variant calling (Figure 2), consistent with previous reports [14]. However, introgression of some mtDNA into the nuclear genomes of sequenced subjects might not be present in the reference genome and would confuse our analysis. Therefore we additionally performed long-range PCR on 72 samples from the whole cohort (36 cases of MDD, 36 controls) to verify that the mtDNA variant sites identified through low-coverage sequencing were not derived from the nuclear genome (Experimental Procedures).

Second, we needed to identify two forms of variation in the mitochondrial genome: mtDNA sequence variants can be present in all copies (homoplasmy) or be present only in a fraction of molecules (heteroplasmy). We defined homoplasmic variants as those sites where there are two alleles present in the cohort, each supported by more than 90% of sequencing reads in individual samples (Experimental Procedures). We identified 1,031 homoplasmic sites occurring at a frequency of greater than 0.1% in our sample, 89% of which were found in 1000 Genomes Phase 3 whole-genome sequencing data [15]. These included all known Asian mitochondrial haplogroups (Supplemental Experimental Procedures; Tables S2 and S3). Using linear regression, no homoplasmic variants were significantly associated with the amount of mtDNA after correction for multiple testing.

We investigated the association between heteroplasmic sites and the amount of mtDNA. As we are more likely to detect heteroplasmy in samples with higher mtDNA coverage (simply because there are more mtDNA reads), we down-sampled coverage of the mtDNA in all samples to exactly 50 reads at each site analyzed. We disregarded sites at which more than 10% of individuals had fewer than 50 reads and therefore could not be down-sampled, so estimates of heteroplasmy from all remaining sites were based on equal coverage on an adequate sample size. To be considered a heteroplasmic site in a sample, we required the presence of two alleles, each supported by two or more reads, and we additionally required the heteroplasmy to be present at a frequency greater than 0.1% (present in more than ten individuals). Using these criteria, we identified 26 heteroplasmic sites from low-coverage sequencing.

In order to ensure that we had accurate estimates not just of the presence but also of the degree of heteroplasmy in an individual, we compared the degree of heteroplasmy called from the ∼100× coverage of mtDNA sequence against that called from ∼500× coverage from PCR amplified mtDNA (in 72 individuals). After considering only those sites where the average degree of heteroplasmy was greater than 10% in both datasets and where the Pearson correlation r^2^ between 100× and 500× coverage data was higher than 0.9, six sites remained suitable for association testing (Supplemental Experimental Procedures; Table S4). One site was significantly associated with mtDNA levels (position = 513, p value = 3.27 × 10^−9^, effect = −0.279, variance explained = 0.48%). This site is known to harbor heteroplasmy and is located in the D-loop regulatory region, but it has not been identified as a transcription factor binding site [14], DNase1-protected site, or splice cut site [16]. We also examined whether the use of down-sampled data might influence our results. Performing the same analysis on the entire dataset led to a decrease in significance of the site at 513 (p value 7.65 × 10^−5^, effect = −0.188, variance explained = 0.15%). The association results for the six heteroplasmic sites with the amount of mtDNA are shown in Table 2.

We explored the relationship between MDD, genetic control over the amount of mtDNA, and heteroplasmy. We asked first whether genetic control over the amount of mtDNA was different in cases of MDD and controls. When we included MDD as a covariate in the GWAS for amount of mtDNA, peaks at TFAM and CDK6 remained significantly associated with amount of mtDNA (p value = 6.96 × 10^−28^, 6.63 × 10^−16^ for rs11006126 and rs445, respectively), and there were no further peaks found. Figure S1 shows the Manhattan and quantile-quantile plots of this GWAS. Testing specifically for an interaction with MDD, we found no region of the genome exceeded genome-wide significance. Interaction p values at the two SNPs that were associated with the amount of mtDNA are 0.70 at rs11006126 and 0.71 at rs445. Therefore, our data indicate effects at both loci on the amount of mtDNA are independent of MDD disease status.

Even though MDD is a strong predictor of the amount of mtDNA [6], and even though the genetic correlation between amount of mtDNA and MDD computed from genome-wide SNPs [8, 17] was 46.7% (SE = 14.3%, p value = 3.53 × 10^−4^), SNPs at TFAM and CDK6 were not associated with MDD (p value = 0.70 for rs11006126, p value = 0.53 for rs445, from a linear mixed model). Furthermore, no homoplasmic variant was associated with MDD in our cohort, nor were any of the six heteroplasmic sites with verified degrees of heteroplasmy associated with MDD (Table S5).

Finally, we asked whether the total amount of heteroplasmy differed between MDD cases and controls. Using the 26 heteroplasmic sites called from low-coverage sequencing on all samples, we computed a heteroplasmic load per sample (number of heteroplasmic sites per sample) in the down-sampled data and found it to be significantly higher in MDD cases (mean fold increase = 1.05, p value = 1.40 × 10^−4^). We performed the same analysis on the high-coverage sequencing of long-range PCR on mtDNA from 72 samples and confirmed our finding: cases of MDD had higher levels of heteroplasmy (p value = 0.032).

**Figure 3:**
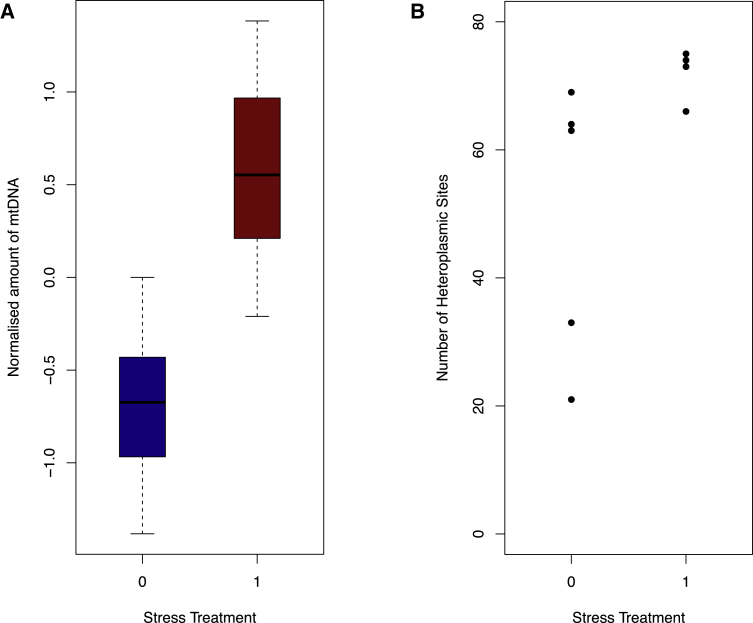
Heteroplasmy Counts in Stressed and Control Mice The figures shows (A) boxplot of the amount of mtDNA quantified from high-coverage sequencing of long-range PCR of mtDNA from liver samples (controlled for starting mass of genomic DNA extracted from mice livers then quantile normalized among all 12 mice) of six female mid-age C56BL/6J mice exposed to a 4-week chronic stress protocol (shown in red), compared to that from the liver samples of six female, mid-age C56BL/6J control mice (shown in blue). Normalized amount of mtDNA from liver samples of six mice was significantly higher than that in non-stressed control mice mean (fold increase = 2.16, p value = 0.0040), and (B) the number of heteroplasmic variant sites found in high-coverage sequencing of long-range PCR of mtDNA (down-sampled to equal coverage of 500 reads per site) from liver samples of six female mid-age C56BL/6J mice exposed to a 4-week chronic stress protocol, compared to that from the liver samples of six female, mid-age C56BL/6J control mice, where each point represents one mouse; stressed mice had significantly higher number of heteroplasmic variants in mtDNA per sample (mean fold increase = 1.46, p value = 0.029).

The human data alone cannot determine whether the increased rates of heteroplasmy are cause or consequence of the stress-related illness; therefore, we turned to an experimental system in which we explored the consequences of exposure to stress in mice [6]. We performed long-range PCR of the mtDNA from 12 female C57BL/6J mice, six of which were subject to a 4-week chronic stress regimen, while six were kept in standard laboratory conditions for the same 4-week period. Stressed mice had higher amounts of mtDNA than controls (mean fold increase = 2.16, p value = 0.004, Figure 3A). We identified 76 heteroplasmic variants in these mice in the same way we did the 72 CONVERGE samples, down-sampling each site to 500 reads. Stressed mice had higher levels of heteroplasmy (mean fold increase = 1.46, p value = 0.029, Figure 3B).

## Discussion

Our study identifies two nuclear genomic loci that contribute to amount of mtDNA and one position in the mitochondrial genome. Of the nuclear loci, TFAM is already known to be essential for mtDNA transcription and is a regulator of mtDNA copy number, replication, and repair [4, 18]. CDK6, by contrast, has not previously been implicated in mitochondrial function (unlike CDK5, which also associates with D-type cyclins [19]).

The association between the amount of mtDNA and a variant in a regulatory region of the mitochondrial genome can be interpreted in two ways: the frequency of the variant may increase as a consequence of increased turnover of mtDNA, or it may itself affect the replication of the mtDNA molecule. While we cannot currently distinguish between these two alternatives, it is worth noting that the association may represent an example of adaptive mutation [20], in which genetic variation occurs in response to the environment, rather than independently of it. Instances of environmentally induced mutations have been documented in bacteria [21], and a similar process may affect the mitochondrial genome.

Intriguingly, none of the variants, either in the nuclear or mitochondrial genome, contribute to the risk of MDD, despite the fact that the genetic correlation between MDD and the amount of mtDNA is 0.46. The latter finding argues that loci exist that exert pleiotropic influence on both risk to depression and mitochondrial function.

Our findings raise questions about how cells manage the turnover of mtDNA sequence. The increase in heteroplasmy we observe in cases of MDD, likely due to stress (as the mouse experiment implies), might appear detrimental to cell survival, given the mutational burden heteroplasmy will impose. However, it should be borne in mind that mtDNA mutations need not damage an organism: indeed, mutations in genes with mitochondrial function can increase lifespan [22]. Intriguingly, overexpression of TFAM also leads to increased cell survival, with improvements at an organ and system level [2, 23, 24, 25, 26]. These effects are due in part, if not entirely, to increases in the number of mitochondria [27], suggesting concerted action between alterations in copy number and sequence to protect cells, and organisms, exposed to stress.

## Experimental Procedures

### Sample Collection, Sequencing, and Genotyping

A description of the sample collection, the low-pass DNA sequencing and genotyping of the nuclear genome is given in [7]. DNA was extracted from saliva samples. mtDNA sequence was processed as described below. All reads mapping to the human mitochondrial genome NC_012920.1 were extracted from the whole genome BAM files mapped to GRCh37.p5 using Samtools (v.0.1.18) [28]. The mitochondrial reads extracted were then converted to the FASTQ format using Picard (v.1.108, http://broadinstitute.github.io/picard/) and mapped to a combined reference containing 894 complete bacterial genomes, 2,024 complete bacterial chromosomes, 154 draft assemblies, and 4,373 complete plasmids sequences (in total 7,390 unique bacterial DNA sequences) available on NCBI using BWA (v.0.5.6) [29]. All reads mapping to bacterial DNA sequences were filtered out using FLAGs attached to each read in the BAM format. Unmapped reads, unpaired reads, reads that did not pass quality control, and reads that may be PCR or optical duplicates were excluded. These procedures filtered out any reads mapping to non-human sequence, as well as removing poor quality reads.

### Estimation of mtDNA Copy Number

We obtained high-coverage mtDNA sequencing reads (102.3×) from paired-end low-coverage (1.7×) whole-genome sequencing data of 10,560 CONVERGE study samples. To quantify mtDNA levels and avoid biases due to potential mapping of similar sequences in nuclear copies of mtDNA (NUMTs) [13] or contaminant bacterial sequences onto the mitochondrial reference, we calculated the mean coverage of mtDNA read pairs per 100 bp along the mtDNA reference both before and after filtering those reads that map uniquely to the human mitochondrial genome reference NC_012920.1 with a high mapping quality filter of 50 (Phred-scale). The intervals with high discrepancies between filtered and unfiltered mappings are those enriched in mtDNA sequences similar to NUMTs or other contaminants, and they are excluded from the calculation of mean mtDNA read depth, which is then controlled for nuclear DNA (chr20) sequencing coverage, sequencing batch, and sample age before transforming to normality by quantile-normalization.

### Estimation of Whole-Genome and Single-Gene SNP-Based Heritability for Amount of mtDNA

Linkage Disequilibrium Adjusted Kinship (LDAK, v.4.9) [8] was used to estimate local linkage disequilibrium (LD) by calculation of local pairwise correlations between SNPs and generating weightings of each SNP in the calculation of a genetic relatedness matrix (GRM) adjusted for local LD between all samples. A GRM was generated using all 6,242,619 SNPs from all chromosomes, on which restricted maximum likelihood (REML) is used for estimating the proportions of variance explained by all SNPs in the GRMs for amount of mtDNA. Gene-based heritability was obtained for all genes using LDAK. Gene boundaries were obtained from RefSeq for human reference genome Build 37, hg19 (February 2009).

### Nuclear DNA GWAS Using a Linear Mixed Model

We performed GWAS on mtDNA levels one chromosome at a time, with a mixed-linear model including a genetic relationship matrix (GRM) constructed from SNPs other than those on the chromosome being studied, and top five PCs from eigen decomposition of the GRM as covariates. This method is implemented in Factored Spectrally Transformed Linear Mixed Models (FastLMM v.2.06.20130802) [30]. For GWAS on mtDNA levels controlling for MDD status, the same linear mixed model was used with input of MDD status of all samples as a binary covariate. Manhattan plots and quantile-quantile plots of the log10 of p values of the GWAS were generated with custom code in R. Genomic-control inflation factor lambda was calculated in R.

### Replication Sample

A measure of mtDNA was obtained from the Avon Longitudinal Study of Parents and Children (ALSPAC) [10] (http://www.bristol.ac.uk/alspac/) whole-genome-sequencing cohort [11], and normalized for the amount of nuclear DNA (using chromosome 20 read depth). Genotype dosages for two SNPs (rs445 and rs1100612) were obtained from ALSPAC and analyzed for association with mtDNA by linear regression, and the joint sample (CONVERGE and ALSPAC) analyzed with a fixed-effects meta-analysis. ALSPAC data are available through a searchable data dictionary (http://www.bris.ac.uk/alspac/researchers/data-access/data-dictionary/). Ethical approval for the study was obtained from the ALSPAC Ethics and Law Committee and the Local Research Ethics Committees.

### Estimation of Genetic Correlation between Amount of mtDNA and MDD

Genetic correlation between amount of mtDNA and MDD was estimated using bivariate REML in GCTA [17] (v.1.24.4), where the input GRM was the same GRM used for estimating whole-genome SNP-based heritability, generated with LDAK using all 6,242,619 SNPs from all chromosomes. The binary MDD status was converted to liability scale with a population prevalence of disease of 8% for this estimation.

### Calling of Homoplasmic and Heteroplasmic Variants in the mtDNA from Low-Coverage Sequencing

We obtained total and per allele read depth for only sequencing reads that map uniquely to the mitochondria using Samtools mpileup (v.0.1.18) [28]. Filtering criteria also include a base quality threshold of 20 (Phred-scaled) for bases and mapping quality of 50 (Phred-scaled) and no more than four mismatches to the reference for reads. To filter out potential mismappings due to nucleotide homopolymer runs on the mtDNA, we only interrogated 14,796 out of 16,569 sites in the mtDNA sequence, masking out 1,773 sites in the mtDNA for being in or beside homopolymer runs of four or more nucleotides.

For calling of homoplasmic variants, we first discarded any site in the mtDNA in any sample that was covered by fewer than ten sequencing reads, and any site that is discarded by this criterion in more than 10% of the samples. A site is considered a homoplasmic variation in the cohort if there were two alleles present in the cohort, and each of them was supported by more than 90% of reads in more than 0.1% of the cohort (ten samples among 10,442). We identified 1,031 homoplasmic sites with the above criteria.

For calling of heteroplasmic variants, sequencing coverage at each site in the mtDNA from each sample was independently and randomly down-sampled to 50 reads; sites in any sample covered by fewer than 50 reads were discarded from the analysis, and sites with more than 10% of the samples discarded by the above criterion were then completely disregarded for further analysis due to lack of evidence. A site was considered potentially heteroplasmic if there was presence of two or more alleles each supported by more than or equal to two reads (4%, out of 50 reads). We then calculated both (1) the degree of heteroplasmy per potential heteroplasmic site in a single individual and (2) the frequency of occurrence of heteroplasmy at a each site in the population. We only considered those sites where more than 0.1% (ten samples among 10,442) of the sample show any degree of heteroplasmy.

### Association Testing with mtDNA Variants Using Linear Regression Model

Testing of association between mtDNA levels and homoplasmic and heteroplasmic variants in the mtDNA above 0.1% in occurrence in CONVERGE were carried out with a linear regression model using a custom script in R. Homoplasmic mtDNA variation among samples was coded as a binary measure representing the reference and alternative alleles relative to the human mitochondrial genome reference NC_012920.1. Heteroplasmic mtDNA variation among samples was coded as residuals from a linear regression of the number of reads supporting the alternative allele with site-specific read depth, whole-genome sequencing coverage and sequencing batch included as covariates. Significance thresholds were determined with a Bonferroni correction for multiple testing on a p value threshold of 0.05.

### Long-Range PCR on mtDNA and High-Coverage Sequencing of PCR Product

For long-range PCR on 72 human DNA samples from CONVERGE, we designed a pair of primers on D-loop sequences on the mtDNA that did not show sequence similarity with nuclear DNA (forward primer: 5′-TGAGGCCAAATATCATTCTGAGGGGC-3′; reverse primer: 5′-TTTCATCATGCGGAGATGTTGGATGG-3′) for the mtDNA. Long-range PCR using these primers covered the whole of the 16,569 bp of the mitochondrial genome. We performed the PCR on 72 samples with thermal cycling conditions as follows: one 1-min cycle at 98°C, 30 cycles of 10 s at 98°C, and then 8 min 15 s at 72°C, one 10-min cycle at 72°C, and then storing PCR products at 4°C.

For long-range PCR on 12 mouse DNA samples, we designed a pair of primers on D-loop sequences on the mtDNA that did not show sequence similarity with nuclear DNA (forward primer: 5′- CCCAGCTACTACCATCATTCAAGT-3′; reverse primer: 5′- GAGAGATTTTATGGGTGTAATGCGG-3′) for the mtDNA. Long-range PCR using these primers covered the whole of the 16,229 bp of the mitochondrial genome. We performed the PCR on 72 samples with thermal cycling conditions as follows: one 1-min cycle at 98°C, 30 cycles of 10 s at 98°C, 30 s at 68°C, and then 8 min 15 s at 72°C, one 10-min cycle at 72°C, and then storing PCR products at 4°C.

PCR mix for both reactions contain 5 μl 5 × GC buffer, 1 μl 10 mM dNTPs, 5 μl Primer cocktail, 0.5 μl 2 U/μl High-Fidelity DNA Polymerase, 100 ng sample DNA.

The PCR products were then sheared to approximately 500-bp fragments and used to construct libraries for sequencing using Illumina Hiseq4000, yielding 100-bp paired-end reads with average per site read depth of 1,757 for human samples and 1,368 for mouse samples. Sequencing products from this process contains only mtDNA.

### Calling of Heteroplasmic Variants in Human and Mouse mtDNA from High-Coverage Sequencing of Long-Range PCR Product

Sequencing reads from human samples were mapped to the human mtDNA reference NC_012920.1 using BWA (v.0.5.6) [29] and Samtools (v.0.1.18) [28], while sequencing reads from mice were mapped using the same software to mtDNA reference sequence in mouse reference genome build GRCm38. We obtained total and per allele read depth for sequencing reads that mapped to the human and mouse mitochondria using Samtools mpileup (v.0.1.18) [28]. Filtering criteria also include a base quality threshold of 20 (Phred-scaled) for bases, and mapping quality of 50 (Phred-scaled) and no more than four mismatches to the reference for reads. To filter out potential mismappings due to nucleotide homopolymer runs on the mtDNA, we only interrogated 14,796 out of 16,569 sites in the human mtDNA sequence and 14,717 out of 16,229 site in the mouse mtDNA sequence, masking out 1,773 and 1,512 sites in the mtDNA for being in or beside homopolymer runs of four or more nucleotides. Sequencing coverage at each site was then independently and randomly down-sampled to 500 reads; sites in any sample covered by fewer than 500 reads were discarded from the analysis, and sites with more than a third of the human and mice samples discarded, respectively, were then completely disregarded for further analysis due to lack of evidence. A site was considered heteroplasmic in a sample if there was presence of two or more alleles each supported by more than or equal to 1% of sequencing reads (five reads out of 500 reads). We then calculated the degree of heteroplasmy per sample per heteroplasmic site.

## Author Contributions

Conceptualization, N.C. and J.F.; Methodology, N.C., S.C., T.M., G.J.H., and J.F.; Validation, N.C., J.L., C.L., X.Z., L.L., Y.C., J.H., and Q.L.; Investigation, N.C., Y.L., S.C., T.M., W.C., and J.F.; Writing – Original Draft, N.C. and J.F; Writing – Review & Editing, N.C., R.M. and J.F.; Funding Acquisition, K.S.K and J.F., Resources, G.J.H., K.S.K., and J.F.; Supervision, J.F.

## Figures and Tables

**Table 1:** Replication of Association with Amount of mtDNA at Top GWAS SNPs

Cohort	SNP	Beta	SE	p
Converge	rs445	0.119	0.015	4.57E-16
ALSPAC	rs445	0.110	0.057	2.14E-02
Joint	rs445	0.118	0.014	3.84E-17
Converge	rs11006126	0.195	0.018	1.17E-27
ALSPAC	rs11006126	0.179	0.047	1.53E-04
Joint	rs11006126	0.193	0.017	1.08E-30

This table shows the effect size (Beta) and its SE and p values (p) from linear regression for association between the two top SNPs (SNP) in *CDK6* (rs445) and *TFAM* (rs11006127) genes in three cohorts (Cohort): our study, CONVERGE; the ALSPAC cohort in UK10K; and a joint cohort containing samples from both CONVERGE and ALSPAC. All associations were significant and the directions of effect at both SNPs in both cohorts are the same. (Note: Linear regression was used for the replication and joint analysis as we did not have whole-genome SNP information from the ALSPAC cohort for a linear mixed-model approach using a GRM, as we did in CONVERGE. The p values for SNP associations with amount of mtDNA in CONVERGE were recalculated with linear regression for comparability with replication and joint analyses.) See also Table S1.

**Table 2:** Association between the Degree of Heteroplasmy at Four Heteroplasmic Sites with the Amount of mtDNA

Marker	Ref.	Alt.	Freq.	Annotation	Gene	Association with mtDNA			
Effect	Var. Exp.	p	Log p
MT146	T	C	0.005	upstream	RNR1,tRNA-Phe	0.650	0.001	6.14E-01	0.212
MT451	A	I	0.002	regulatory	NA	−0.501	0.000	4.76E-01	0.322
MT513	G	I	0.416	regulatory	NA	−0.279	0.005	3.27E-09	8.485
MT5894	A	I	0.017	regulatory	NA	−0.617	0.001	3.36E-01	0.473
MT15939	C	I	0.014	regulatory	NA	−0.005	0.000	9.45E-01	0.025
MT16129	G	A	0.020	upstream;downstream	tRNA-Pro;CYTB,tRNA-Thr	−0.167	0.000	6.36E-01	0.197

This table shows the association between degree of heteroplasmy at four heteroplasmic sites in the mtDNA and amount of mtDNA quantified from low-coverage sequencing in 10,442 samples from CONVERGE. The first four columns show the position of the heteroplasmic site in mtDNA (Marker), reference allele in the human mitochondrial genome reference NC_012920.1 (Ref.), the alternative allele (Alt.) for which the heteroplasmy is detected, and the frequency of occurrence of this heteroplasmy in the cohort (Freq.). An “I” in the Alt. column means the heteroplasmy is for an insertion or deletion mutation. The next two columns show characteristics of the four heteroplasmic sites: annotation of variant function (Annotation) and nearest gene (Gene). The final four columns show the results of association testing between degree of heteroplasmy at each site with amount of mtDNA by linear regression: direction of effect (Effect) from linear regression, variance of amount of mtDNA explained by the site heteroplasmy (Var. Exp.) from difference in residual sum of squares in ANOVA between the model with and without degree of heteroplasmy as the test term in linear regression, p value of association (p) between degree of heteroplasmy and amount of mtDNA, and –log10 of the p value (Log p). One site position 513 is significantly associated with amount of mtDNA, with a positive effect, and it lies in the D-loop regulatory region in the mtDNA. See also Table S5.

## Supplemental Information

Document S1. Supplemental Experimental Procedures, Figure S1, and Tables S1–S5

Document S2. Article plus Supplemental Information
