# Supplementary material for: Genetic Control over mtDNA and Its Relationship to Major Depressive Disorder
Source: Curr Biol. 2015 Dec 21;25(24):3170–7. doi: 10.1016/j.cub.2015.10.065 (PMC4691240; doi:10.1016/j.cub.2015.10.065)
Supplement: Document S1. Supplemental Experimental Procedures, Figure S1, and Tables S1–S5 [file mmc1.pdf]

Current Biology
Supplemental Information

Genetic Control over mtDNA and Its Relationship to Major Depressive Disorder

Na Cai, Yihan Li , Simon Chang, Jieqin Liang, Chongyun Lin, Xiufei Zhang, Lu Liang, Jingchu Hu, Wharton Chan, Kenneth S Kendler, Tomas Malinauskas, Guo-Jen Huang, Qibin Li, Richard Mott, and Jonathan Flint

Figure S1, Related to Figure1: GWAS on mtDNA with MDD as covariate

A

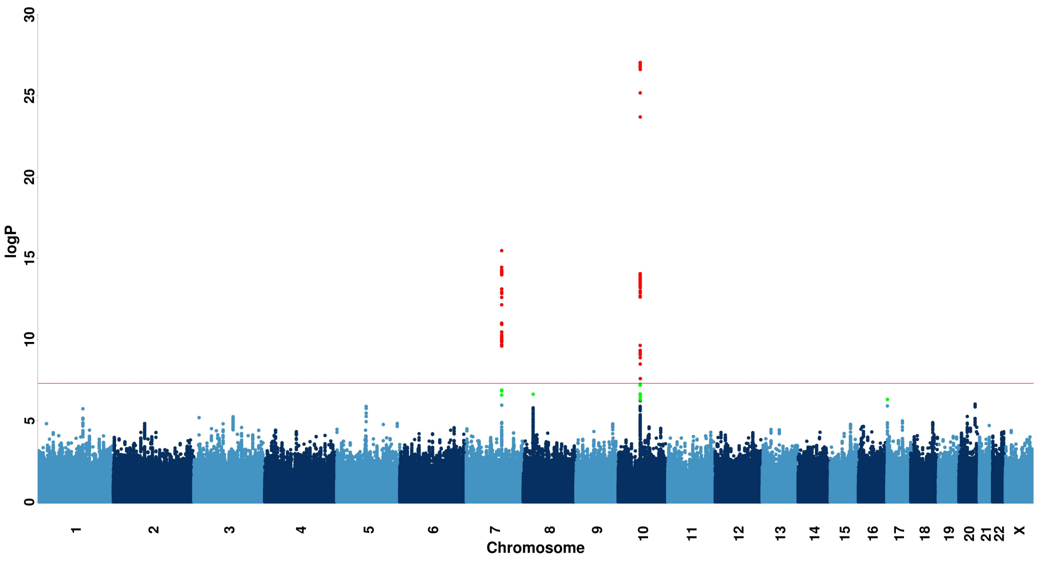

B

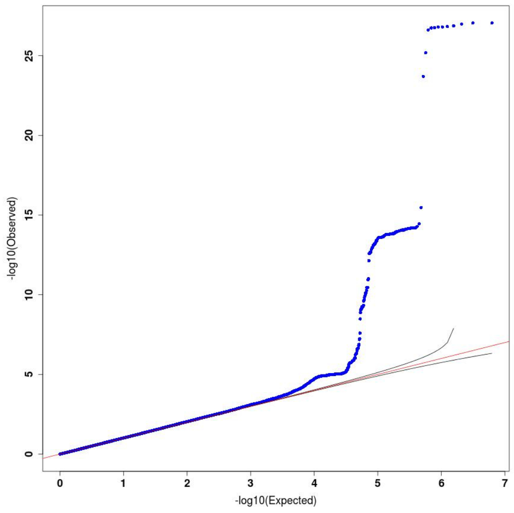

(A) Manhattan plot of genome wide association for amount of mtDNA with MDD as covariate. (B) Quantile-quantile plot of GWAS for amount of mtDNA with MDD as covariate, using leave-one-chromosome-out (LOCO) linear mixed model implemented in FastLMM on 10,442 samples (5,224 cases of MDD, 5,218 controls), genomic control lambda (λ) = 1.017.

Table S1, Related to Table 1: List of SNPs associated with amount of mtDNA with P values < 10^{-6}

CHR	POS	RSID	REF	ALT	FREQ	INFO	HWE_P	P	LOGP
7	92058506	rs10267476	C	T	0.319	0.911	1.20E-06	6.90E-07	6.161
7	92266198	rs147826719	C	T	0.321	0.963	1.20E-01	1.45E-12	11.840
7	92290278	rs41459146	T	C	0.327	0.986	2.40E-01	5.97E-13	12.224
7	92295417	rs2282985	C	G	0.502	0.992	4.90E-01	2.06E-09	8.686
7	92296668	rs56353205	T	C	0.326	0.988	2.30E-01	3.24E-13	12.489
7	92296829	rs4727280	C	T	0.480	0.978	6.60E-01	1.24E-09	8.907
7	92299545	rs2282986	T	C	0.327	0.993	2.80E-01	2.28E-13	12.643
7	92299964	rs2106135	C	T	0.327	0.993	2.80E-01	2.22E-13	12.653
7	92300863	rs2301557	C	T	0.326	0.988	2.50E-01	2.79E-13	12.555
7	92301040	rs9640606	C	T	0.495	0.993	7.10E-01	8.64E-10	9.063
7	92303196	rs10254702	A	G	0.502	0.993	6.70E-01	1.17E-09	8.934
7	92303554	rs3757823	A	G	0.502	0.992	6.80E-01	9.35E-10	9.029
7	92305445	rs75882441	T	A	0.328	0.992	4.20E-01	1.88E-13	12.725
7	92305515	rs6977712	A	T	0.483	0.991	7.10E-01	5.49E-10	9.261
7	92305899	rs6973871	T	C	0.483	0.991	7.40E-01	5.49E-10	9.260
7	92308798	rs12670783	A	G	0.502	0.991	6.70E-01	7.55E-10	9.122
7	92312803	rs2282987	G	C	0.329	0.989	5.80E-01	1.46E-14	13.837
7	92313733	rs2237573	A	G	0.500	0.994	7.10E-01	2.92E-10	9.535
7	92315330	rs6964803	C	T	0.328	0.996	4.20E-01	1.35E-14	13.868
7	92315660	rs11981340	T	C	0.329	0.997	4.90E-01	1.61E-14	13.792
7	92315774	rs11981374	T	C	0.327	0.992	4.60E-01	6.73E-15	14.172
7	92316244	rs13437843	C	T	0.329	0.998	5.00E-01	1.65E-14	13.782
7	92316282	rs60726864	T	A	0.329	0.998	5.00E-01	1.65E-14	13.782
7	92317374	rs2282988	T	C	0.329	0.998	4.90E-01	1.97E-14	13.706
7	92317887	rs10243384	C	T	0.477	0.997	7.70E-01	5.94E-11	10.226
7	92319472	rs11533993	T	C	0.329	0.998	4.90E-01	1.74E-14	13.760
7	92319488	rs11533994	A	C	0.501	0.994	6.30E-01	1.85E-10	9.733
7	92320169	rs10267477	T	C	0.501	0.996	6.00E-01	1.82E-10	9.739
7	92321584	rs1972508	G	A	0.588	0.939	1.90E-02	1.02E-10	9.993
7	92324050	rs11981129	C	T	0.483	0.997	6.00E-01	5.10E-11	10.293
7	92324355	rs10225660	T	C	0.328	0.996	5.10E-01	1.34E-14	13.873
7	92324419	rs17164721	G	T	0.328	0.996	5.40E-01	1.41E-14	13.850
7	92326346	rs2282989	C	T	0.327	0.997	5.30E-01	9.87E-15	14.005
7	92328956	rs1004051	C	A	0.327	0.997	4.70E-01	1.17E-14	13.932
7	92329126	rs3731318	C	T	0.327	0.997	4.70E-01	1.17E-14	13.931
7	92329957	rs1004052	C	G	0.327	0.997	4.90E-01	1.29E-14	13.888
7	92330480	rs3802079	C	T	0.327	0.997	4.70E-01	1.17E-14	13.931
7	92331695	rs2079146	T	C	0.480	0.995	6.70E-01	4.40E-11	10.357
7	92333408	rs10254840	A	G	0.477	0.981	8.80E-01	1.38E-10	9.859
7	92408370	rs445	C	T	0.342	0.906	1.70E-03	6.03E-16	15.219
7	92428239	rs2299242	C	G	0.408	0.923	3.90E-03	5.53E-07	6.257
7	92464778	rs78065312	C	G	0.228	0.903	4.20E-06	3.16E-10	9.500

7	92484093	rs10953073	A	G	0.336	0.910	4.70E-06	3.18E-07	6.497
7	92489813	rs2023699	A	G	0.336	0.910	5.30E-06	3.19E-07	6.496
8	15607313	rs2543154	G	A	0.511	0.984	6.80E-01	2.52E-07	6.598
10	59827743	rs61851889	G	A	0.145	0.997	6.80E-01	9.21E-07	6.036
10	59829407	rs2221296	C	T	0.145	0.998	7.60E-01	8.78E-07	6.057
10	59830413	rs61851890	C	G	0.145	0.998	7.90E-01	9.22E-07	6.035
10	59837321	rs61851911	G	A	0.145	0.999	7.90E-01	9.42E-07	6.026
10	59839893	rs61851913	G	C	0.145	0.998	7.90E-01	9.42E-07	6.026
10	59844227	rs80306558	G	T	0.160	0.991	8.30E-01	3.84E-08	7.416
10	59849616	rs16911771	C	G	0.160	0.988	7.50E-01	4.67E-08	7.331
10	59854651	rs1698469	A	C	0.808	0.987	5.90E-02	7.60E-07	6.119
10	59855663	rs61850576	A	T	0.162	0.987	8.10E-01	4.53E-08	7.344
10	59862105	rs61850577	G	T	0.167	0.979	7.30E-01	2.01E-10	9.697
10	59870890	rs61850578	C	T	0.145	0.992	8.50E-01	5.93E-10	9.227
10	59874268	rs16911810	T	A	0.145	0.992	8.20E-01	5.93E-10	9.227
10	59876328	rs77599563	G	T	0.145	0.992	7.90E-01	5.77E-10	9.239
10	59880706	rs61850584	G	T	0.145	0.989	7.60E-01	4.80E-10	9.319
10	59889478	rs138825781	T	C	0.145	0.992	7.60E-01	4.86E-10	9.313
10	59891501	rs12569763	C	A	0.145	0.991	7.90E-01	4.86E-10	9.314
10	59904889	rs73287122	A	G	0.149	0.994	9.10E-01	1.28E-13	12.893
10	59905514	rs61850589	G	T	0.149	0.994	9.10E-01	1.28E-13	12.893
10	59905849	rs10509086	C	G	0.150	0.995	9.70E-01	1.42E-13	12.846
10	59906486	rs73287129	G	A	0.150	0.995	1.00E+00	1.42E-13	12.846
10	59908601	rs61850590	A	G	0.150	0.993	1.00E+00	1.25E-13	12.904
10	59914787	rs12571364	G	A	0.151	0.993	1.00E+00	3.14E-14	13.503
10	59925048	rs7072206	C	T	0.151	0.992	4.90E-01	2.07E-14	13.683
10	59927787	rs57851212	A	T	0.152	0.993	4.90E-01	1.71E-14	13.766
10	59929700	rs77599079	T	C	0.152	0.992	5.90E-01	5.82E-15	14.235
10	59938336	rs12570088	A	G	0.155	0.994	5.00E-01	6.15E-15	14.211
10	59941791	rs10509088	C	T	0.156	0.999	9.40E-01	6.40E-15	14.194
10	59946672	rs1199105	T	C	0.156	0.999	9.20E-01	1.25E-14	13.902
10	59947543	rs12252749	A	T	0.156	0.999	9.40E-01	1.13E-14	13.948
10	59957000	rs77350161	G	A	0.156	0.995	9.40E-01	1.09E-14	13.963
10	59957071	rs12252003	A	C	0.149	0.938	2.10E-01	7.73E-15	14.112
10	59960083	rs1625716	T	G	0.155	0.998	8.90E-01	1.11E-14	13.957
10	59966533	rs58375950	C	T	0.155	0.998	8.30E-01	1.21E-14	13.918
10	59967203	rs60679872	C	T	0.155	0.999	8.60E-01	1.21E-14	13.918
10	59969111	rs12261547	G	C	0.155	0.999	8.30E-01	1.21E-14	13.918
10	59970430	rs61875332	G	T	0.155	0.999	8.60E-01	1.21E-14	13.918
10	59971818	rs12251970	C	T	0.155	0.998	8.60E-01	1.21E-14	13.917
10	59974730	rs61875333	G	C	0.155	0.998	8.00E-01	1.21E-14	13.916
10	59987662	rs12572236	G	T	0.155	0.998	8.60E-01	8.60E-15	14.065
10	59990325	rs11006087	C	T	0.155	0.998	8.00E-01	8.60E-15	14.065
10	60001675	rs7900114	C	T	0.155	0.998	8.00E-01	1.01E-14	13.994
10	60004329	rs139600710	G	C	0.155	0.995	6.20E-01	8.62E-15	14.064
10	60023959	rs61873887	G	A	0.155	0.998	8.60E-01	8.33E-15	14.079

10	60031460	rs12572520	T	G	0.155	0.998	7.80E-01	8.22E-15	14.085
10	60032636	rs2790176	A	G	0.155	0.998	7.80E-01	8.22E-15	14.085
10	60035152	rs2275442	C	T	0.155	0.998	7.80E-01	8.22E-15	14.085
10	60047177	rs3758567	G	C	0.155	0.999	8.00E-01	1.21E-14	13.917
10	60049790	rs12241783	G	T	0.155	0.999	7.50E-01	1.01E-14	13.997
10	60052201	rs11006105	C	T	0.155	0.999	7.50E-01	1.01E-14	13.997
10	60052999	rs12264744	C	T	0.155	0.999	7.50E-01	1.01E-14	13.997
10	60065855	rs59030640	G	A	0.154	0.990	8.60E-01	7.57E-15	14.121
10	60084394	rs112187169	C	A	0.151	0.985	4.80E-01	9.81E-14	13.008
10	60092695	rs61873953	G	A	0.156	0.994	2.40E-01	8.43E-15	14.074
10	60095105	rs112660736	G	T	0.137	0.958	5.00E-01	9.84E-15	14.007
10	60095266	rs16912153	G	A	0.137	0.958	5.00E-01	9.86E-15	14.006
10	60099333	rs59474782	G	A	0.146	0.942	6.00E-03	9.23E-14	13.035
10	60100089	rs4948291	C	T	0.158	0.994	4.80E-01	1.83E-14	13.738
10	60106835	rs1963927	G	T	0.462	0.996	5.00E-01	1.27E-07	6.895
10	60116030	rs12255735	G	C	0.156	0.993	2.30E-01	7.47E-15	14.127
10	60118681	rs10826172	T	C	0.462	0.996	5.40E-01	1.13E-07	6.947
10	60121976	rs11006120	C	G	0.158	0.993	4.60E-01	1.19E-14	13.926
10	60122066	rs11006121	C	T	0.463	0.991	3.30E-01	1.14E-07	6.943
10	60122678	rs4145785	A	T	0.462	0.996	5.00E-01	1.16E-07	6.937
10	60125877	rs11006122	C	T	0.462	0.996	4.80E-01	1.14E-07	6.942
10	60127410	rs10826174	G	C	0.464	0.989	4.40E-01	1.06E-07	6.974
10	60142116	rs9971282	C	T	0.162	0.956	6.40E-02	2.39E-27	26.622
10	60142402	rs11006125	A	T	0.168	0.982	8.30E-02	1.11E-27	26.957
10	60142800	rs9971104	G	T	0.168	0.984	9.00E-02	9.16E-28	27.038
10	60142880	rs11006126	T	C	0.169	0.978	9.00E-02	8.73E-28	27.059
10	60144207	rs4390300	G	A	0.489	0.987	1.40E-03	7.22E-07	6.141
10	60144884	rs2279340	C	T	0.171	0.990	3.50E-01	2.76E-27	26.558
10	60144998	rs2279339	C	A	0.170	0.992	3.50E-01	2.74E-27	26.563
10	60145079	rs12247015	A	G	0.173	0.979	3.90E-01	1.99E-27	26.701
10	60145342	rs1937	G	C	0.170	0.989	4.10E-01	2.47E-27	26.608
10	60145597	rs4397793	G	C	0.494	0.984	2.70E-03	7.02E-07	6.154
10	60147270	rs10826178	G	A	0.491	0.997	2.60E-03	6.80E-07	6.168
10	60147784	rs10763537	G	A	0.491	0.997	3.00E-03	6.24E-07	6.205
10	60148692	rs2306604	A	G	0.492	0.993	2.50E-03	5.79E-07	6.238
10	60151026	rs10826179	A	G	0.492	0.996	1.90E-03	6.00E-07	6.222
10	60155120	rs1049432	G	T	0.172	0.995	4.90E-01	2.95E-27	26.530
10	60156166	rs11006132	A	G	0.172	0.995	5.10E-01	2.83E-27	26.548
10	60156584	rs11006133	G	A	0.491	0.993	2.30E-03	5.68E-07	6.246
10	60157339	rs7089361	C	T	0.491	0.992	2.10E-03	5.47E-07	6.262
10	60158385	rs12259591	G	A	0.489	0.997	1.60E-03	6.11E-07	6.214
10	60158446	rs12254586	T	C	0.489	0.997	1.80E-03	6.12E-07	6.213
10	60159362	rs12245545	T	G	0.489	0.996	1.60E-03	6.15E-07	6.211
10	60160312	rs7077225	G	T	0.490	0.991	2.60E-03	6.42E-07	6.192
10	60160455	rs16912212	A	G	0.489	0.996	1.30E-03	6.04E-07	6.219
10	60161172	rs4525176	G	A	0.491	0.994	1.00E-03	4.97E-07	6.304

10	60161640	rs10763540	A	G	0.491	0.994	1.20E-03	4.97E-07	6.304
10	60164560	rs11006137	G	A	0.499	0.984	1.10E-03	1.54E-07	6.813
10	60164820	rs10826182	C	T	0.499	0.984	1.10E-03	1.59E-07	6.798
10	60167882	rs6481387	C	T	0.499	0.984	2.30E-03	1.45E-07	6.840
10	60168003	rs7905675	G	A	0.499	0.984	2.30E-03	1.45E-07	6.840
10	60169880	rs11817790	G	T	0.499	0.983	2.50E-03	1.48E-07	6.830
10	60175440	rs61190999	C	T	0.172	0.962	4.50E-01	2.96E-25	24.529
10	60193608	rs76455223	T	A	0.171	0.933	9.40E-01	1.38E-23	22.861
10	60228964	rs61875518	G	A	0.157	0.967	1.20E-01	1.12E-13	12.952
10	60229347	rs1427215	G	A	0.157	0.970	5.60E-02	1.37E-13	12.862
10	60230671	rs61875520	C	G	0.160	0.989	3.80E-01	1.51E-13	12.820
10	60231657	rs7093118	T	C	0.160	0.989	3.80E-01	1.51E-13	12.820
10	60240354	rs17644676	A	G	0.161	0.990	4.80E-01	2.15E-13	12.669
10	60241036	rs35258735	T	A	0.161	0.990	4.80E-01	2.15E-13	12.669
10	60244111	rs61875523	A	G	0.161	0.990	4.80E-01	2.16E-13	12.666
10	60245124	rs77192138	G	A	0.156	0.983	5.90E-01	4.60E-13	12.337
10	60247792	rs61875524	C	T	0.124	0.997	2.30E-01	2.03E-09	8.693
10	60257846	rs74335736	G	A	0.124	0.998	2.90E-01	2.28E-09	8.642
10	60258420	rs77095691	T	C	0.124	0.998	2.90E-01	2.28E-09	8.642
10	60261176	rs76383270	C	T	0.124	0.998	2.90E-01	2.28E-09	8.642
10	60261367	rs76110033	A	T	0.124	0.998	2.90E-01	2.28E-09	8.642
10	60262181	rs78111290	T	C	0.124	0.998	2.90E-01	2.28E-09	8.642
10	60264332	rs1427208	T	A	0.124	0.991	3.60E-01	4.94E-09	8.307
10	60264609	rs11006167	G	A	0.127	0.972	3.40E-01	8.44E-09	8.073
10	60293094	rs12571046	A	C	0.107	0.936	4.90E-01	4.91E-08	7.309
10	60374087	rs11006205	A	C	0.107	0.945	6.30E-01	7.99E-07	6.097

This table shows the list of SNPs associated with amount of mtDNA quantified from 1.7X coverage next generation sequencing on 10,442 samples with P values < 10^{-6} in a leave-one-chromosome-out linear-mixed model implemented in FastLMM. The first five columns contain the chromosome (CHR), position (POS), SNP identifier (RSID), and reference (REF) and alternative (ALT) alleles; the next three columns show the alternative allele frequency (FREQ) of the SNPs in CONVERGE, the IMPUTE2-style imputation information score (IMPUTE2-INFO) of the imputed allele dosages, and the P-value of violation of Hardy Weinberg Equilibrium (HWE_P); the last two columns show the P-value of association of the SNPs with amount of mtDNA in CONVERGE in a linear mixed-model (P) and the –log10 of the P-value (LOGP).

Table S2, Related to Experimental Procedures: Frequency of diagnostic alleles for mitochondrial haplogroups

Haplogroup	Diagnostic site	Reference Allele	Diagnostic Allele	Frequency (%)					
				Central Asia	East Asia	Han Chinese	1000G	1000G CHSCHB	CONVERGE
A	663	A	G	7.00	7.00	4.00-16.70	5.54	6.02	7.21
B	8280-8289	ACCCCCTCTA	A	5.00	16.00	0.60-3.30	NA	NA	NA
C	13263	A	G	12.00	5.00	2.00-8.00	3.04	3.24	3.98
D	5178	C	A	15.00	26.00	1.40-3.30	5.15	22.55	21.67
E	13626	C	T	0.00	0.00	NA	NA	NA	0.09
F	6392	T	C	5.00	11.00	1.40-6.00	3.71	14.42	14.34
G	4833	A	G	5.00	4.00	NA	0.00	0.00	14.77
H	7028	C	C	15.00	1.00	NA	11.37	0.00	NA
I	4529	A	T	1.00	0.00	NA	0.05	0.00	NA
J	13708	G	A	3.00	1.00	NA	5.27	5.56	4.31
K	12308	A	G	1.00	0.00	NA	NA	NA	NA
L	3594	C	T	0.00	0.00	NA	15.78	0.00	0.02
S	8404	T	C	0.00	0.00	NA	0.19	0.92	0.28
T	4917	A	G	6.00	0.00	NA	0.22	0.00	0.28
U	12308	A	G	10.00	0.00	NA	NA	NA	NA
V	4580	G	A	0.00	0.00	NA	NA	NA	NA
W	11947	A	G	2.00	0.00	NA	0.96	0.00	0.06
X	6371	C	T	0.00	0.00	NA	0.43	0.00	0.02
Y	8392	G	A	1.00	1.00	1.30-3.80	0.19	0.00	NA
Z	9090	T	C	2.00	2.00	1.30-7.10	0.31	1.85	3.24

This table shows in the first four columns the mitochondrial haplogroup, diagnostic diagnostic site for the haplogroup, the reference allele in the human mtDNA reference NC_012920.1, and the diagnostic allele. The next six columns show the frequency (%) of occurrence of the diagnostic allele in Central Asians and East Asians from MitoMap[S1], estimates from multiple studies in Han Chinese population[S2], all 1000 Genomes Phase 3 samples, 1000 Genomes Phase 3 Chinese Beijing (CHB) and Chinese South (CHS) samples, and CONVERGE study samples.

Table S3, Related to Experimental Procedures: Correlation between PC1 in CONVERGE and four homoplasmic variants adaptive to arctic climate

Variant position	Adaptive allele	Haplogroup	Gene	Amino acid change	Linear regression P value with PC1	Coefficient	Allele frequency
4824	G	A	ND2	T112A	NA	NA	NA
8794	T	A	ATP6	H90Y	NA	NA	NA
11969	A	C	ND4	A404T	0.00056	0.0022	0.020
15204	C	C	cytb	I153T	0.00290	0.0025	0.013

This table shows in the first three columns position, adaptive allele, haplogroup in which four previously identified homoplasmic variants reported to be present in Asian mitochondrial Haplogroups (A, C, D and X) [S3] and associated with colder climates. The next two columns show the genes which the variants reside in, and the amino acid changes they cause. The next two columns show the association P value and regression coefficient between the presence of the adaptive alleles and PC1 in CONVERGE (Experimental Procedures), which captures the North-South cline. The final column shows the allele frequency of the adaptive alleles in CONVERGE. Two sites at positions 4824 and 8794 were not called in CONVERGE due to lack of coverage.

Table S4, Related to Experimental Procedures: Correlation between degree of heteroplasmy called in 72 samples from low coverage sequencing and high-coverage sequencing of long range PCR of mtDNA

Position	Mean degree of heteroplasmy		Pearson r2	P-value
	Low coverage	Long range PCR		
**MT146**	**0.118**	**0.126**	**0.999**	**0.000**
**MT451**	**0.011**	**0.012**	**0.999**	**0.000**
MT512	0.000	0.006	NA	NA
**MT513**	**0.374**	**0.411**	**0.984**	**0.000**
MT567	0.004	0.011	0.999	0.000
MT955	0.000	0.002	NA	NA
MT1290	0.000	0.000	NA	NA
MT2226	0.000	0.000	NA	NA
MT2487	0.010	0.000	0.013	0.340
MT3167	0.000	0.000	NA	NA
MT3243	0.000	0.000	NA	NA
MT3447	0.000	0.001	NA	NA
**MT5894**	**0.033**	**0.060**	**0.829**	**0.000**
MT6289	0.000	0.001	NA	NA
MT10283	0.000	0.000	0.003	0.663
MT10306	0.027	0.000	0.000	0.942
MT11031	0.001	0.005	0.000	0.911
MT11866	0.000	0.002	NA	NA
MT12417	0.002	0.005	0.013	0.341
MT12684	0.000	0.000	0.004	0.615
MT15536	0.000	0.000	NA	NA
MT15900	0.000	0.000	0.001	0.806
**MT15939**	**0.012**	**0.012**	**1.000**	**0.000**
**MT16129**	**0.183**	**0.183**	**0.993**	**0.000**
MT16179	0.003	0.057	0.003	0.657
MT16496	0.002	0.092	0.017	0.285

This table shows the degrees of heteroplasmy in 72 samples for whom there were both low-coverage sequencing data and high coverage sequencing data from long range PCR of mtDNA. The first column shows the 26 sites in the mtDNA identified to be heteroplasmic at an occurrence of higher than 0.1% in CONVERGE. The next two columns show the mean degree of heteroplasmy among the 72 samples at each of the 26 sites, quantified from low coverage sequencing and high coverage sequencing of long range PCR product on mtDNA respectively. Where the mean degree of heteroplasmy is 0 in either columns, there was no heteroplasmy detected in any of the 72 samples using the relevant dataset. The next two columns show the per site Pearson correlation r2 and

between the degree of heteroplasmy called from low coverage sequencing and high coverage sequencing of long range PCR on mtDNA in 72 sample, and the P-value of the correlation. Six sites highlighted in bold showed mean degrees of heteroplasmy of over 1% in both datasets and were highly correlated, and were included in association testing between site-specific degree of heteroplasmy and amount of mtDNA.

Table S5, Related to Table 3: Association between degree of heteroplasmy at six heteroplasmic sites with MDD

MARKER	REF	ALT	FREQ	TYPE	GENE	MDD			
						EFFECT	VAREXP	P	LOGP
MT146	T	C	0.005	upstream	RNR1,tRNA-Phe	1.275	0.003	2.39E-01	0.622
MT451	A	I	0.002	regulatory	NA	0.734	0.000	8.39E-02	1.076
MT513	G	I	0.416	regulatory	NA	-0.035	0.000	2.31E-01	0.636
MT5894	A	I	0.017	regulatory	NA	-0.582	0.001	3.15E-01	0.502
MT15939	C	I	0.014	regulatory	NA	0.051	0.000	1.66E-01	0.780
MT16129	G	A	0.020	upstream;downstream	tRNA-Pro;CYTB,tRNA-Thr	-0.229	0.000	2.16E-01	0.665

This table shows the association between degree of heteroplasmy at four heteroplasmic sites in the mtDNA and major depressive disorder (MDD) in 10,442 samples. The first four columns show the position of the heteroplasmic site in mtDNA (MARKER), reference allele in the human mitochondrial genome reference NC_012920.1 (REF), the alternative allele (ALT) for which the heteroplasmy is detected, and the frequency of occurrence of this heteroplasmy in the cohort (FREQ). An "I" in the ALT column means the heteroplasmy is for an insertion or deletion mutation. The next two columns show characterisitics of the four heteroplasmic sites: annotation of variant function (ANNOTATION) and nearest gene (GENE). The final four columns show the results of association testing between degree of heteroplasmy at each site with amount of mtDNA by logistic regression: direction of effect (EFFECT) from linear regression, variance of amount of mtDNA explained by the site heteroplasmy (VAREXP) from difference in residual sum of squares in analysis of variance (ANOVA) between the model with and without degree of heteroplasmy as the test term in logistic regression, P value of association (P) between degree of heteroplasmy and MDD, and –log10 of the P value (LOGP). Degree of heteroplasmy at none of the sites were significantly associated with MDD.

Supplemental Experimental Procedures

1. Verifying homoplasmic mtDNA variation calls

As accurate calls of mtDNA homoplasmic variants form the basis of association analyses and are important in preventing misidentification of heteroplasmic variants in the mtDNA, we checked for both sensitivity and accuracy in calling homoplasmic variants from low-coverage whole-genome sequencing data. The strategy to call homoplasmic variants was to look for non-reference alleles supported by more than 90% of the reads at any particular site in each sample, but only at those sites where there are more than 10 uniquely mapped reads of high mapping quality (>59 in Phred scale).

Sensitivity of variant calling

To estimate the sensitivity of our variant calling method, we applied our method of homoplasmic variant calling on the 1000 Genomes Phase 3 whole-genome sequencing data (n=2,598, of which 216 are from Han Chinese populations, CHB and CHS). We compared the set of common homoplasmic variants (>0.1%) we called in CONVERGE with homoplasmic variants called in 1000 Genomes Project Phase 3 samples. 948 out of 1,030 (88.85%) of the common homoplasmic variants we found in CONVERGE were found to be polymorphic in 1000 Genomes Phase 3, and 546 of them (51.17%) were found to be occurring at >0.1% frequency in the Han Chinese populations.

Presence of mtDNA haplogroup markers

We checked the allele frequencies of homoplasmic variant sites called in CONVERGE at diagnostic SNPs for haplogroups against previously documented frequencies in Central Asians and East Asians in MitoMap [S1], the combined estimates of allele frequencies from previous studies on Han Chinese populations [S2], as well as allele frequencies called from all 1000 Genomes Phase 3 samples and those from Han Chinese populations (Table S2). The frequencies of alleles at SNPs diagnostic for the different haplogroups are highly consistent with those previously reported.

We checked for the presence of four homoplasmic variants previously reported to be present in Asian mitochondrial Haplogroups (A, C, D and X) [S3] and associated with colder climates in our sample. Two of the four variants were identified as homoplasmic

variants occurring at frequencies above 0.1% in our sample, and were found to be associated with PC1, capturing the North-South cline of geographical origin of our samples, calculated from eigen decomposition of the a genetic relatedness matrix calculated from 322,911 common SNPs of minor allele frequency (MAF) > 1% and linkage disequilibrium $r^2 < 0.5$ using GCTA (version 1.24.4, Methods)[S4] (Table S3). Two variants were not called in our dataset due to lack of coverage at the sites.

2. Verifying heteroplasmic mtDNA variation calls by long range polymerase chain reaction (PCR) and sequencing of mitochondrial DNA

To identify and quantify the number of heteroplasmic sites independent of sequence coverage we down-sampled the mtDNA reads so that each site was covered by 50 reads. We disregarded sites at which more than 10% of individuals did not fulfill this criterion, so that estimates of heteroplasmy from all remaining sites were based on equal coverage on an adequate sample size. We required the presence of two alleles, each supported by two or more reads at sites and occurring at higher than 0.1% frequency in the cohort (10 individuals), for a site to be considered heteroplasmic. Using these criteria we identified heteroplasmy at 26 positions in the mtDNA.

Low-coverage sequencing has a high false-negative rate in the calling of heteroplasmic sites, and some of the heteroplasmies we detected using low-coverage sequencing might be reads originating from NUMTs mapping incorrectly to the mtDNA reference. Therefore we performed the same analysis using the high-coverage sequencing on 72 samples we obtained with long-range PCR, ensuring both high-coverage and mtDNA origin of sequencing reads.

We quantified heteroplasmy in the 72 samples, after down-sampling the high-coverage sequencing to 500 reads per site and discarding those samples with fewer than 500 reads covering at each site. We only counted heteroplasmic sites where the fraction of reads supporting the minor heteroplasmic allele was higher than 1% (supported by a minimum of 5 reads). This yielded 408 such sites. Comparison between mean degree of heteroplasmy obtained from low-coverage sequencing on 72 samples and high-coverage sequencing of products of long range PCR on mtDNA on the same samples showed that when the mean degree of heteroplasmy among samples is high (10% or higher) in both low-coverage sequencing and long range PCR data, the correlation between the two estimates is high (Table S4). For lower degrees of heteroplasmy, this correlation drops substantially. Therefore we considered further only sites where heteroplasmy rates are greater than 10% in an individual.

Supplemental References

S1. Lott, M.T., et al., *mtDNA Variation and Analysis Using MITOMAP and MITOMASTER.* Curr Protoc Bioinformatics, 2013. **1**(123): p. 1 23 1-1 23 26.

S2. Yao, Y.G., et al., *Phylogeographic differentiation of mitochondrial DNA in Han Chinese.* Am J Hum Genet, 2002. **70**(3): p. 635-51.

S3. Ruiz-Pesini, E., et al., *Effects of purifying and adaptive selection on regional variation in human mtDNA.* Science, 2004. **303**(5655): p. 223-6.

S4. Yang, J., et al., *GCTA: a tool for genome-wide complex trait analysis.* Am J Hum Genet, 2011. **88**(1): p. 76-82.
