# Supplementary material for: Genetic Control over mtDNA and Its Relationship to Major Depressive Disorder
Source: Curr Biol. 2015 Dec 21;25(24):3170–7. doi: 10.1016/j.cub.2015.10.065 (PMC4691240; doi:10.1016/j.cub.2015.10.065)
Supplement: Document S2. Article plus Supplemental Information [file mmc2.pdf]

Current Biology

Article

Genetic Control over mtDNA and Its Relationship to Major Depressive Disorder

Highlights

- Loci near TFAM and CDK6 contribute to variation in the amount of mtDNA
- Mutations accumulate in the mtDNA of cases of major depression
- Animal experiments show that heteroplasmy can be induced by chronic stress
- Amount of mtDNA is associated with site-specific heteroplasmy

Authors

Na Cai, Yihan Li, Simon Chang, ..., Qibin Li, Richard Mott, Jonathan Flint

Correspondence

jf@well.ox.ac.uk

In Brief

Having previously shown that cases of major depressive disorder have increased mtDNA, that exposure to stress is likely responsible, and that the changes in amount of mtDNA are reversible, Cai et al. investigate in this paper the genetic control and consequences of this change and how this relates to major depressive disorder.

Cai et al., 2015, Current Biology *25*, 3170–3177
December 21, 2015 ©2015 The Authors
http://dx.doi.org/10.1016/j.cub.2015.10.065

Genetic Control over mtDNA and Its Relationship to Major Depressive Disorder

Na Cai,[1] Yihan Li,[1] Simon Chang,[2] Jieqin Liang,[3] Chongyun Lin,[3] Xiufei Zhang,[3] Lu Liang,[3] Jingchu Hu,[3] Wharton Chan,[1] Kenneth S. Kendler,[4] Tomas Malinauskas,[5] Guo-Jen Huang,[2] Qibin Li,[3] Richard Mott,[1] and Jonathan Flint[1,*]
[1]Wellcome Trust Centre for Human Genetics, University of Oxford, Roosevelt Drive, Oxford, Oxfordshire OX3 7BN, UK
[2]Department and Graduate Institute of Biomedical Sciences, College of Medicine, Chang Gung University, Tao-Yuan 33302, Taiwan
[3]BGI-Shenzhen, Floor 9 Complex Building, Beishan Industrial Zone, Yantian District, Shenzhen, Guangdong 518083, China
[4]Virginia Institute for Psychiatric and Behavioral Genetics, Virginia Commonwealth University, Richmond, VA 23298, USA
[5]Cold Spring Harbor Laboratory, Beckman Building, One Bungtown Road, Cold Spring Harbor, NY 11724, USA
*Correspondence: jf@well.ox.ac.uk
http://dx.doi.org/10.1016/j.cub.2015.10.065
This is an open access article under the CC BY license (http://creativecommons.org/licenses/by/4.0/).

SUMMARY

Control over the number of mtDNA molecules per cell appears to be tightly regulated, but the mechanisms involved are largely unknown. Reversible alterations in the amount of mtDNA occur in response to stress suggesting that control over the amount of mtDNA is involved in stress-related diseases including major depressive disorder (MDD). Using low-coverage sequence data from 10,442 Chinese women to compute the normalized numbers of reads mapping to the mitochondrial genome as a proxy for the amount of mtDNA, we identified two loci that contribute to mtDNA levels: one within the *TFAM* gene on chromosome 10 (rs11006126, p value = 8.73×10^{-28}, variance explained = 1.90%) and one over the *CDK6* gene on chromosome 7 (rs445, p value = 6.03×10^{-16}, variance explained = 0.50%). Both loci replicated in an independent cohort. *CDK6* is thus a new molecule involved in the control of mtDNA. We identify increased rates of heteroplasmy in women with MDD, and show from an experimental paradigm using mice that the increase is likely due to stress. Furthermore, at least one heteroplasmic variant is significantly associated with changes in the amount of mtDNA (position 513, p value = 3.27×10^{-9}, variance explained = 0.48%) suggesting site-specific heteroplasmy as a possible link between stress and increase in amount of mtDNA. These findings indicate the involvement of mitochondrial genome copy number and sequence in an organism's response to stress.

INTRODUCTION

The number of mtDNA molecules appears to be tightly regulated, as inferred from the constant amount of mtDNA per mitochondrion in different cells in the presence of marked variation in the number of mitochondria between cell types [1]. The mechanisms involved are largely unknown [2, 3]. One of the components of the mtDNA replication machinery, mitochondrial transcription factor A (*TFAM*), appears to be a key transcription factor controlling the amount of mtDNA [4], but it is still not known how that signal is used by cells to count the amount of mtDNA that they require. The observation that the number of mitochondria predicts cell division better than other measures such as cell volume or cell size [5] suggests replication of mtDNA and mitochondrial biogenesis, while autonomous, may be regulated by cell-cycle machinery and vice versa.

We recently observed that the amount of mtDNA alters in response to external stress: there was significantly more mtDNA in the saliva and blood of people with major depression than in controls [6]. Chronic stress also altered the amount of mtDNA in mouse tissues, and, at least in part, was restored to pre-stress levels following cessation of stress [6]. Changes in cellular composition could not account for these observations [6], suggesting that the mtDNA alterations reflected changes within cells, and corticosteroid signaling down the hypothalamic-pituitary axis may be involved in causing these changes since injection of corticosterone alone can recapitulate effects of chronic stress.

These findings raised questions as to what contributes to changes in the amount of mtDNA, how that is related to major depressive disorder, and what are the consequences for a cell of maintaining a high turnover of mtDNA molecules, which have relatively high mutation rates. We set out to use genotypes and measures of mtDNA levels from whole-genome low-coverage sequencing on 10,442 Han Chinese women recruited in the CONVERGE (China, Oxford, and Virginia Commonwealth University Experimental Research on Genetic Epidemiology) Consortium of Major Depressive Disorder (MDD). Using this dataset, we set out to perform the first genome-wide association study (GWAS) on mtDNA levels to discover what might be involved in the molecular pathways controlling mtDNA levels. We further ask whether variation in mtDNA sequences contributes to variations in the amount of mtDNA and whether such variation is related to MDD.

RESULTS

CONVERGE obtained sequence from Chinese women with a mean coverage of 1.7× of the nuclear genome [7]. For this study,

CrossMark

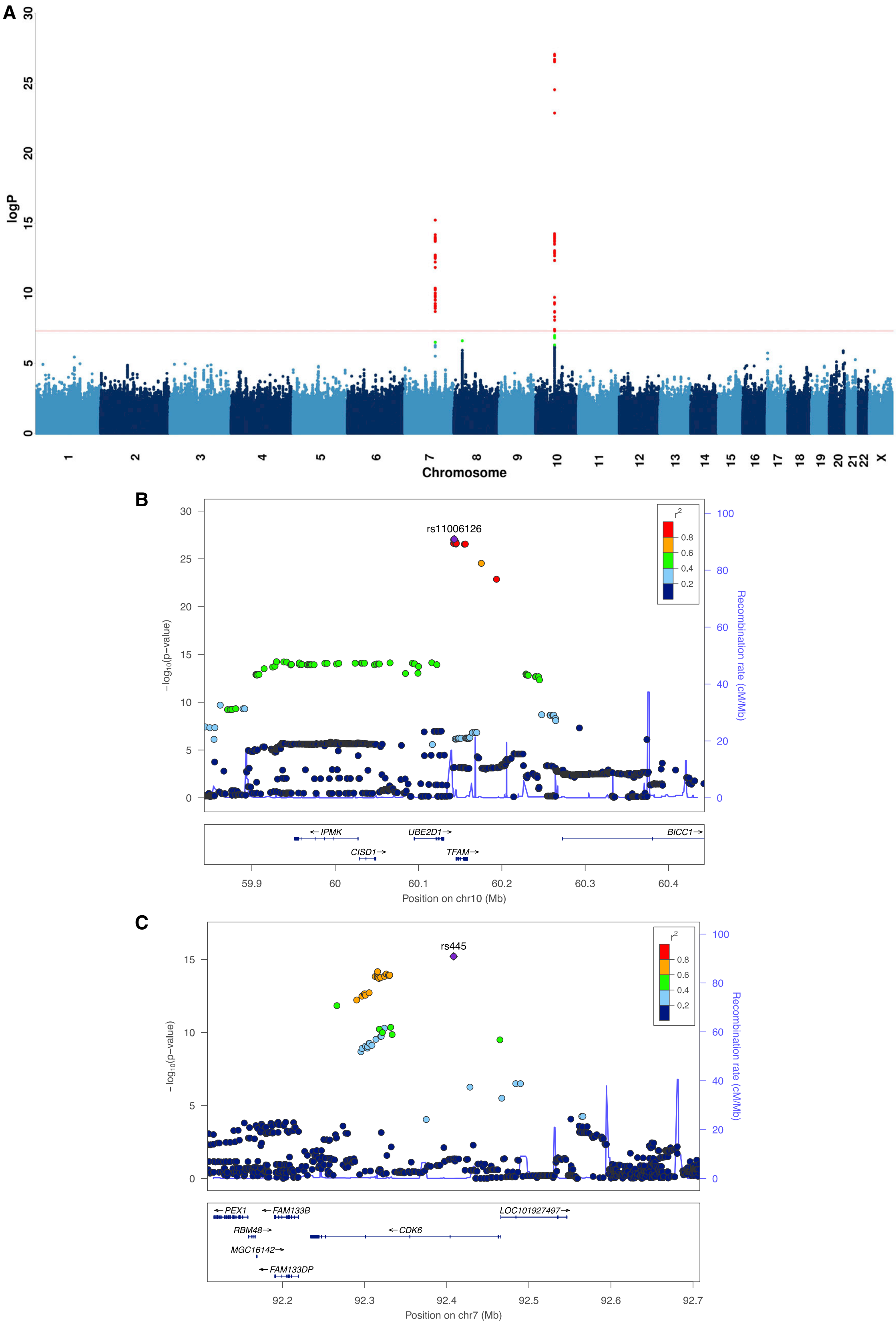

(legend on next page)

Table 1. Replication of Association with Amount of mtDNA at Top GWAS SNPs

Cohort	SNP	Beta	SE	p
Converge	rs445	0.119	0.015	4.57E-16
ALSPAC	rs445	0.110	0.057	2.14E-02
Joint	rs445	0.118	0.014	3.84E-17
Converge	rs11006126	0.195	0.018	1.17E-27
ALSPAC	rs11006126	0.179	0.047	1.53E-04
Joint	rs11006126	0.193	0.017	1.08E-30

This table shows the effect size (Beta) and its SE and p values (p) from linear regression for association between the two top SNPs (SNP) in *CDK6* (rs445) and *TFAM* (rs11006127) genes in three cohorts (Cohort): our study, CONVERGE; the ALSPAC cohort in UK10K; and a joint cohort containing samples from both CONVERGE and ALSPAC. All associations were significant and the directions of effect at both SNPs in both cohorts are the same. (Note: Linear regression was used for the replication and joint analysis as we did not have whole-genome SNP information from the ALSPAC cohort for a linear mixed-model approach using a GRM, as we did in CONVERGE. The p values for SNP associations with amount of mtDNA in CONVERGE were recalculated with linear regression for comparability with replication and joint analyses.) See also Table S1.

we used sequences from 5,224 women with MDD and 5,218 controls, and imputed allele dosages at 6,242,619 SNPs across the nuclear genome (details on imputation methodology and genotype quality are given in [7]). We began by determining that the amount of mtDNA, defined as the number of reads mapping to the mitochondrial genome over read coverage of the nuclear genome, controlled for age of subject and sequencing batch (Experimental Procedures), is a heritable trait, with an estimated SNP-based heritability [8] of 15.6% (SE = 5.1%; p value = 1.06×10^{-3}). We then mapped variation in the amount of mtDNA as a quantitative trait and identified two loci that exceeded genome-wide significance (genomic control lambda [λ] = 1.017; Figure 1A). One locus is within the *TFAM* gene on chromosome 10 (top SNP rs11006126, p value = 8.73×10^{-28}, variance explained by gene = 1.90%, Figure 1B), and the second lies over the *CDK6* gene on chromosome 7 (top SNP rs445, p value = 6.03×10^{-16}, variance explained by gene = 0.50%, Figure 1C).

The most highly associated SNP (rs11006126) resides in the 3′ region of the gene *TFAM*. The most highly associated SNPs at the *CDK6* locus (rs445) lie in intron 1 of the *CDK6* gene. Both associations replicated in a cohort of 1,753 samples from the Avon Longitudinal Study of Parent and Children (ALSPAC) [10], that forms part of the UK10K study [11]. Notably DNA from this cohort was obtained from blood. Association results for the top two SNPs (rs445 and rs11006126) in separate and joint analyses are summarized in Table 1. All SNPs associated with the amount of mtDNA in the CONVERGE study at p values $<10^{-6}$ are shown in Table S1. Our measure of the amount of mtDNA from sequencing data is highly correlated with that from another recently published method [12]. GWAS using measures from both methods of quantification gave highly similar results (data not shown).

We next asked whether the mtDNA sequence itself governs the alterations in mtDNA quantity, and whether cycles of replication might result in mutations in the mtDNA molecule. The latter was considered likely given the relatively higher mutation rate of mtDNA compared to genomic DNA. We therefore set out to identify variant sites in the 16 Kb mitochondrial genome and to quantify their frequency both between and within individuals (coverage of each individual's mitochondrial genome is approximately 100-fold).

Analysis of variants in mtDNA is subject to a number of potential confounds, which we took care to avoid. First, we had to ensure that the sequence variants we obtained were truly in the mitochondrial and not in the nuclear genome (since the latter contains partial copies of the former [13]), and also that they were not derived from exogenous sources (DNA for this project was extracted from saliva). After applying stringent quality-control filters and criteria (Experimental Procedures), 89% of the mitochondrial genome was considered sufficiently unique for variant calling (Figure 2), consistent with previous reports [14]. However, introgression of some mtDNA into the nuclear genomes of sequenced subjects might not be present in the reference genome and would confuse our analysis. Therefore we additionally performed long-range PCR on 72 samples from the whole cohort (36 cases of MDD, 36 controls) to verify that the mtDNA variant sites identified through low-coverage sequencing were not derived from the nuclear genome (Experimental Procedures).

Second, we needed to identify two forms of variation in the mitochondrial genome: mtDNA sequence variants can be present in all copies (homoplasmy) or be present only in a fraction of molecules (heteroplasmy). We defined homoplasmic variants as those sites where there are two alleles present in the cohort, each supported by more than 90% of sequencing reads in individual samples (Experimental Procedures). We identified 1,031 homoplasmic sites occurring at a frequency of greater than 0.1% in our sample, 89% of which were found in 1000 Genomes Phase 3 whole-genome sequencing data [15]. These included all known Asian mitochondrial haplogroups (Supplemental Experimental Procedures; Tables S2 and S3). Using linear regression, no homoplasmic variants were significantly associated with the amount of mtDNA after correction for multiple testing.

We investigated the association between heteroplasmic sites and the amount of mtDNA. As we are more likely to detect heteroplasmy in samples with higher mtDNA coverage (simply because there are more mtDNA reads), we down-sampled coverage of the mtDNA in all samples to exactly 50 reads at each site analyzed. We disregarded sites at which more than 10% of individuals had fewer than 50 reads and therefore could not be down-sampled, so estimates of heteroplasmy from all

Figure 1. Two Loci Associated with mtDNA

Manhattan plot of genome-wide association for amount of mtDNA (A). Detailed views of two loci associated with amount of mtDNA over the *TFAM* region on chromosome 10 at 60.1 Mb (B) and the *CDK6* gene on chromosome 7 at position 92.4 Mb (C) are shown. The −log10 p values of imputed SNPs associated with amount of mtDNA are shown on the left y axis. The horizontal axis gives chromosomal position in megabases (Mb). Genes within the regions are shown in the bottom panels. Linkage disequilibrium of each SNP with top SNP, shown in large purple diamond, is indicated by its color. The plots were drawn using LocusZoom [9]. See also Figure S1.

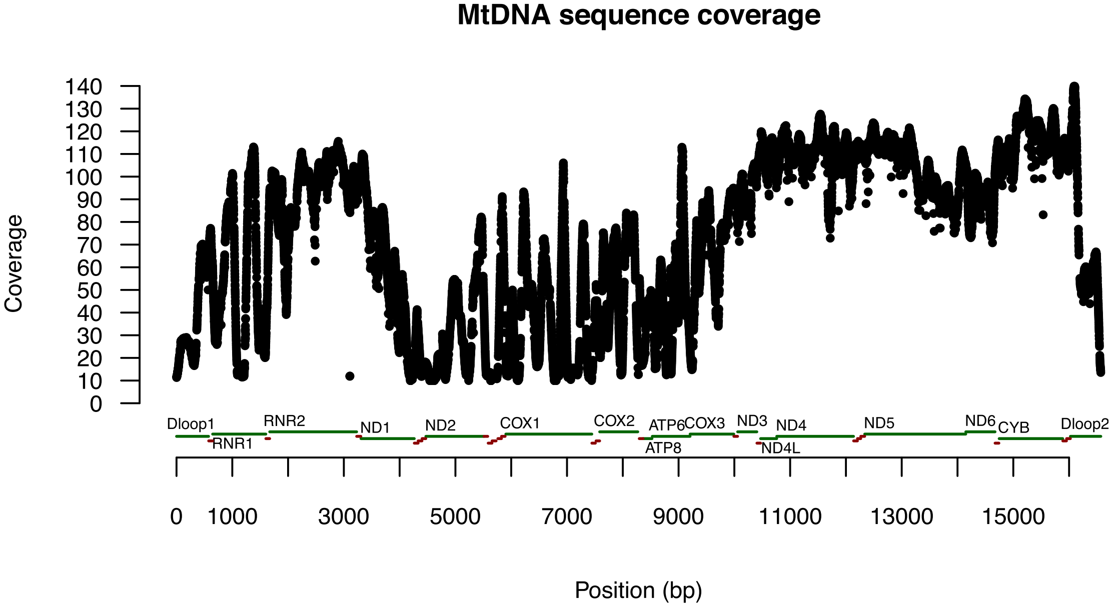

Figure 2. Accessibility of mtDNA for Variant Calling

Mean per site read depth across 10,442 samples for all sites on the mtDNA. Reads included in the per site read count have passed the following quality control criteria: (1) mapping quality >59, (2) both ends of paired-end read map uniquely to the mtDNA reference NC_012920.1, (3) they do not contain mismatches to the mtDNA reference with total length of 5 bp or above, and (4) base at site in question has base quality of higher than 20. Sites with more than 10% samples with read depth less than 10 would not be used for calling homoplasmic variants, and sites with more than 10% samples with read depth less than 50 would not be used for calling heteroplasmic variants. See also Experimental Procedures.

remaining sites were based on equal coverage on an adequate sample size. To be considered a heteroplasmic site in a sample, we required the presence of two alleles, each supported by two or more reads, and we additionally required the heteroplasmy to be present at a frequency greater than 0.1% (present in more than ten individuals). Using these criteria, we identified 26 heteroplasmic sites from low-coverage sequencing.

In order to ensure that we had accurate estimates not just of the presence but also of the degree of heteroplasmy in an individual, we compared the degree of heteroplasmy called from the ~100× coverage of mtDNA sequence against that called from ~500× coverage from PCR amplified mtDNA (in 72 individuals). After considering only those sites where the average degree of heteroplasmy was greater than 10% in both datasets and where the Pearson correlation r^2 between 100× and 500× coverage data was higher than 0.9, six sites remained suitable for association testing (Supplemental Experimental Procedures; Table S4). One site was significantly associated with mtDNA levels (position = 513, p value = 3.27×10^{-9}, effect = −0.279, variance explained = 0.48%). This site is known to harbor heteroplasmy and is located in the D-loop regulatory region, but it has not been identified as a transcription factor binding site [14], DNase1-protected site, or splice cut site [16]. We also examined whether the use of down-sampled data might influence our results. Performing the same analysis on the entire dataset led to a decrease in significance of the site at 513 (p value 7.65×10^{-5}, effect = −0.188, variance explained = 0.15%). The association results for the six heteroplasmic sites with the amount of mtDNA are shown in Table 2.

We explored the relationship between MDD, genetic control over the amount of mtDNA, and heteroplasmy. We asked first whether genetic control over the amount of mtDNA was different in cases of MDD and controls. When we included MDD as a covariate in the GWAS for amount of mtDNA, peaks at TFAM and CDK6 remained significantly associated with amount of mtDNA (p value = 6.96×10^{-28}, 6.63×10^{-16} for rs11006126 and rs445, respectively), and there were no further peaks found. Figure S1 shows the Manhattan and quantile-quantile plots of this GWAS. Testing specifically for an interaction with MDD, we found no region of the genome exceeded genome-wide significance. Interaction p values at the two SNPs that were associated with the amount of mtDNA are 0.70 at rs11006126 and 0.71 at rs445. Therefore, our data indicate effects at both loci on the amount of mtDNA are independent of MDD disease status.

Even though MDD is a strong predictor of the amount of mtDNA [6], and even though the genetic correlation between amount of mtDNA and MDD computed from genome-wide SNPs [8, 17] was 46.7% (SE = 14.3%, p value = 3.53×10^{-4}), SNPs at TFAM and CDK6 were not associated with MDD (p value = 0.70 for rs11006126, p value = 0.53 for rs445, from a linear mixed model). Furthermore, no homoplasmic variant was associated with MDD in our cohort, nor were any of the six heteroplasmic sites with verified degrees of heteroplasmy associated with MDD (Table S5).

Finally, we asked whether the total amount of heteroplasmy differed between MDD cases and controls. Using the 26 heteroplasmic sites called from low-coverage sequencing on all samples, we computed a heteroplasmic load per sample (number of heteroplasmic sites per sample) in the down-sampled data and found it to be significantly higher in MDD cases (mean fold increase = 1.05, p value = 1.40×10^{-4}). We performed the same analysis on the high-coverage sequencing

Table 2. Association between the Degree of Heteroplasmy at Four Heteroplasmic Sites with the Amount of mtDNA

						Association with mtDNA			
Marker	Ref.	Alt.	Freq.	Annotation	Gene	Effect	Var. Exp.	p	Log p
MT146	T	C	0.005	upstream	RNR1,tRNA-Phe	0.650	0.001	6.14E-01	0.212
MT451	A	I	0.002	regulatory	NA	−0.501	0.000	4.76E-01	0.322
MT513	G	I	0.416	regulatory	NA	−0.279	0.005	3.27E-09	8.485
MT5894	A	I	0.017	regulatory	NA	−0.617	0.001	3.36E-01	0.473
MT15939	C	I	0.014	regulatory	NA	−0.005	0.000	9.45E-01	0.025
MT16129	G	A	0.020	upstream;downstream	tRNA-Pro;CYTB,tRNA-Thr	−0.167	0.000	6.36E-01	0.197

This table shows the association between degree of heteroplasmy at four heteroplasmic sites in the mtDNA and amount of mtDNA quantified from low-coverage sequencing in 10,442 samples from CONVERGE. The first four columns show the position of the heteroplasmic site in mtDNA (Marker), reference allele in the human mitochondrial genome reference NC_012920.1 (Ref.), the alternative allele (Alt.) for which the heteroplasmy is detected, and the frequency of occurrence of this heteroplasmy in the cohort (Freq.). An "I" in the Alt. column means the heteroplasmy is for an insertion or deletion mutation. The next two columns show characteristics of the four heteroplasmic sites: annotation of variant function (Annotation) and nearest gene (Gene). The final four columns show the results of association testing between degree of heteroplasmy at each site with amount of mtDNA by linear regression: direction of effect (Effect) from linear regression, variance of amount of mtDNA explained by the site heteroplasmy (Var. Exp.) from difference in residual sum of squares in ANOVA between the model with and without degree of heteroplasmy as the test term in linear regression, p value of association (p) between degree of heteroplasmy and amount of mtDNA, and –log10 of the p value (Log p). One site position 513 is significantly associated with amount of mtDNA, with a positive effect, and it lies in the D-loop regulatory region in the mtDNA. See also Table S5.

of long-range PCR on mtDNA from 72 samples and confirmed our finding: cases of MDD had higher levels of heteroplasmy (p value = 0.032).

The human data alone cannot determine whether the increased rates of heteroplasmy are cause or consequence of the stress-related illness; therefore, we turned to an experimental system in which we explored the consequences of exposure to stress in mice [6]. We performed long-range PCR of the mtDNA from 12 female C57BL/6J mice, six of which were subject to a 4-week chronic stress regimen, while six were kept in standard laboratory conditions for the same 4-week period. Stressed mice had higher amounts of mtDNA than controls (mean fold increase = 2.16, p value = 0.004, Figure 3A). We identified 76 heteroplasmic variants in these mice in the same way we did the 72 CONVERGE samples, down-sampling each site to 500 reads. Stressed mice had higher levels of heteroplasmy (mean fold increase = 1.46, p value = 0.029, Figure 3B).

DISCUSSION

Our study identifies two nuclear genomic loci that contribute to amount of mtDNA and one position in the mitochondrial genome. Of the nuclear loci, TFAM is already known to be essential for mtDNA transcription and is a regulator of mtDNA copy number, replication, and repair [4, 18]. CDK6, by contrast, has not previously been implicated in mitochondrial function (unlike CDK5, which also associates with D-type cyclins [19]).

The association between the amount of mtDNA and a variant in a regulatory region of the mitochondrial genome can be interpreted in two ways: the frequency of the variant may increase as a consequence of increased turnover of mtDNA, or it may itself affect the replication of the mtDNA molecule. While we cannot currently distinguish between these two alternatives, it is worth noting that the association may represent an example of adaptive mutation [20], in which genetic variation occurs in response to the environment, rather than independently of it. Instances of environmentally induced mutations have been documented in bacteria [21], and a similar process may affect the mitochondrial genome.

Intriguingly, none of the variants, either in the nuclear or mitochondrial genome, contribute to the risk of MDD, despite the fact that the genetic correlation between MDD and the amount of mtDNA is 0.46. The latter finding argues that loci exist that exert pleiotropic influence on both risk to depression and mitochondrial function.

Our findings raise questions about how cells manage the turnover of mtDNA sequence. The increase in heteroplasmy we observe in cases of MDD, likely due to stress (as the mouse experiment implies), might appear detrimental to cell survival, given the mutational burden heteroplasmy will impose. However, it should be borne in mind that mtDNA mutations need not damage an organism: indeed, mutations in genes with mitochondrial function can increase lifespan [22]. Intriguingly, overexpression of TFAM also leads to increased cell survival, with improvements at an organ and system level [2, 23–26]. These effects are due in part, if not entirely, to increases in the number of mitochondria [27], suggesting concerted action between alterations in copy number and sequence to protect cells, and organisms, exposed to stress.

EXPERIMENTAL PROCEDURES

Sample Collection, Sequencing, and Genotyping

A description of the sample collection, the low-pass DNA sequencing and genotyping of the nuclear genome is given in [7]. DNA was extracted from saliva samples. mtDNA sequence was processed as described below. All reads mapping to the human mitochondrial genome NC_012920.1 were extracted from the whole genome BAM files mapped to GRCh37.p5 using Samtools (v.0.1.18) [28]. The mitochondrial reads extracted were then converted to the FASTQ format using Picard (v.1.108, http://broadinstitute.github.io/picard/) and mapped to a combined reference containing 894 complete bacterial genomes, 2,024 complete bacterial chromosomes, 154 draft assemblies, and 4,373 complete plasmids sequences (in total 7,390 unique bacterial DNA sequences) available on NCBI using BWA (v.0.5.6) [29]. All reads mapping to bacterial DNA sequences were filtered out using FLAGs attached to each read in the BAM format. Unmapped reads, unpaired reads, reads that did not pass quality control, and reads that may be PCR or optical duplicates

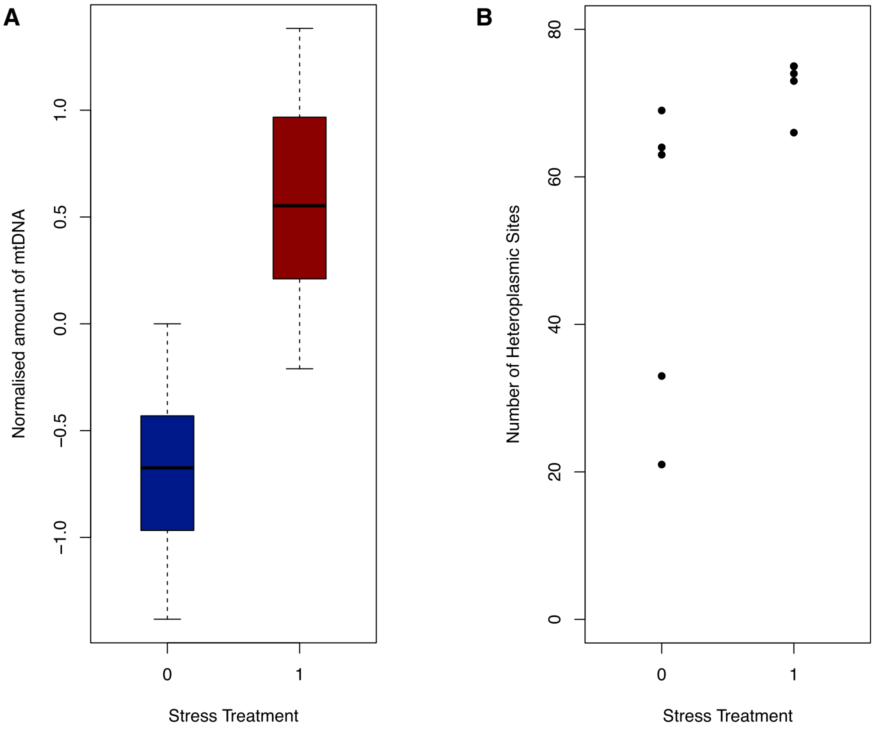

Figure 3. Heteroplasmy Counts in Stressed and Control Mice

The figures shows (A) boxplot of the amount of mtDNA quantified from high-coverage sequencing of long-range PCR of mtDNA from liver samples (controlled for starting mass of genomic DNA extracted from mice livers then quantile normalized among all 12 mice) of six female mid-age C56BL/6J mice exposed to a 4-week chronic stress protocol (shown in red), compared to that from the liver samples of six female, mid-age C56BL/6J control mice (shown in blue). Normalized amount of mtDNA from liver samples of six mice was significantly higher than that in non-stressed control mice mean (fold increase = 2.16, p value = 0.0040), and (B) the number of heteroplasmic variant sites found in high-coverage sequencing of long-range PCR of mtDNA (down-sampled to equal coverage of 500 reads per site) from liver samples of six female mid-age C56BL/6J mice exposed to a 4-week chronic stress protocol, compared to that from the liver samples of six female, mid-age C56BL/6J control mice, where each point represents one mouse; stressed mice had significantly higher number of heteroplasmic variants in mtDNA per sample (mean fold increase = 1.46, p value = 0.029).

were excluded. These procedures filtered out any reads mapping to non-human sequence, as well as removing poor quality reads.

Estimation of mtDNA Copy Number

We obtained high-coverage mtDNA sequencing reads (102.3×) from paired-end low-coverage (1.7×) whole-genome sequencing data of 10,560 CONVERGE study samples. To quantify mtDNA levels and avoid biases due to potential mapping of similar sequences in nuclear copies of mtDNA (NUMTs) [13] or contaminant bacterial sequences onto the mitochondrial reference, we calculated the mean coverage of mtDNA read pairs per 100 bp along the mtDNA reference both before and after filtering those reads that map uniquely to the human mitochondrial genome reference NC_012920.1 with a high mapping quality filter of 50 (Phred-scale). The intervals with high discrepancies between filtered and unfiltered mappings are those enriched in mtDNA sequences similar to NUMTs or other contaminants, and they are excluded from the calculation of mean mtDNA read depth, which is then controlled for nuclear DNA (chr20) sequencing coverage, sequencing batch, and sample age before transforming to normality by quantile-normalization.

Estimation of Whole-Genome and Single-Gene SNP-Based Heritability for Amount of mtDNA

Linkage Disequilibrium Adjusted Kinship (LDAK, v.4.9) [8] was used to estimate local linkage disequilibrium (LD) by calculation of local pairwise correlations between SNPs and generating weightings of each SNP in the calculation of a genetic relatedness matrix (GRM) adjusted for local LD between all samples. A GRM was generated using all 6,242,619 SNPs from all chromosomes, on which restricted maximum likelihood (REML) is used for estimating the proportions of variance explained by all SNPs in the GRMs for amount of mtDNA. Gene-based heritability was obtained for all genes using LDAK. Gene boundaries were obtained from RefSeq for human reference genome Build 37, hg19 (February 2009).

Nuclear DNA GWAS Using a Linear Mixed Model

We performed GWAS on mtDNA levels one chromosome at a time, with a mixed-linear model including a genetic relationship matrix (GRM) constructed from SNPs other than those on the chromosome being studied, and top five PCs from eigen decomposition of the GRM as covariates. This method is implemented in Factored Spectrally Transformed Linear Mixed Models (FastLMM v.2.06.20130802) [30]. For GWAS on mtDNA levels controlling for MDD status, the same linear mixed model was used with input of MDD status of all samples as a binary covariate. Manhattan plots and quantile-quantile plots of the log10 of p values of the GWAS were generated with custom code in R. Genomic-control inflation factor lambda was calculated in R.

Replication Sample

A measure of mtDNA was obtained from the Avon Longitudinal Study of Parents and Children (ALSPAC) [10] (http://www.bristol.ac.uk/alspac/) whole-genome-sequencing cohort [11], and normalized for the amount of nuclear DNA (using chromosome 20 read depth). Genotype dosages for two SNPs (rs445 and rs1100612) were obtained from ALSPAC and analyzed for association with mtDNA by linear regression, and the joint sample (CONVERGE and ALSPAC) analyzed with a fixed-effects meta-analysis. ALSPAC data are available through a searchable data dictionary (http://www.bris.ac.uk/alspac/researchers/data-access/data-dictionary/). Ethical approval for the study was obtained from the ALSPAC Ethics and Law Committee and the Local Research Ethics Committees.

Estimation of Genetic Correlation between Amount of mtDNA and MDD

Genetic correlation between amount of mtDNA and MDD was estimated using bivariate REML in GCTA [17] (v.1.24.4), where the input GRM was the same GRM used for estimating whole-genome SNP-based heritability, generated with LDAK using all 6,242,619 SNPs from all chromosomes. The binary MDD status was converted to liability scale with a population prevalence of disease of 8% for this estimation.

Calling of Homoplasmic and Heteroplasmic Variants in the mtDNA from Low-Coverage Sequencing

We obtained total and per allele read depth for only sequencing reads that map uniquely to the mitochondria using Samtools mpileup (v.0.1.18) [28]. Filtering criteria also include a base quality threshold of 20 (Phred-scaled) for bases and mapping quality of 50 (Phred-scaled) and no more than four

mismatches to the reference for reads. To filter out potential mismappings due to nucleotide homopolymer runs on the mtDNA, we only interrogated 14,796 out of 16,569 sites in the mtDNA sequence, masking out 1,773 sites in the mtDNA for being in or beside homopolymer runs of four or more nucleotides.

For calling of homoplasmic variants, we first discarded any site in the mtDNA in any sample that was covered by fewer than ten sequencing reads, and any site that is discarded by this criterion in more than 10% of the samples. A site is considered a homoplasmic variation in the cohort if there were two alleles present in the cohort, and each of them was supported by more than 90% of reads in more than 0.1% of the cohort (ten samples among 10,442). We identified 1,031 homoplasmic sites with the above criteria.

For calling of heteroplasmic variants, sequencing coverage at each site in the mtDNA from each sample was independently and randomly down-sampled to 50 reads; sites in any sample covered by fewer than 50 reads were discarded from the analysis, and sites with more than 10% of the samples discarded by the above criterion were then completely disregarded for further analysis due to lack of evidence. A site was considered potentially heteroplasmic if there was presence of two or more alleles each supported by more than or equal to two reads (4%, out of 50 reads). We then calculated both (1) the degree of heteroplasmy per potential heteroplasmic site in a single individual and (2) the frequency of occurrence of heteroplasmy at a each site in the population. We only considered those sites where more than 0.1% (ten samples among 10,442) of the sample show any degree of heteroplasmy.

Association Testing with mtDNA Variants Using Linear Regression Model

Testing of association between mtDNA levels and homoplasmic and heteroplasmic variants in the mtDNA above 0.1% in occurrence in CONVERGE were carried out with a linear regression model using a custom script in R. Homoplasmic mtDNA variation among samples was coded as a binary measure representing the reference and alternative alleles relative to the human mitochondrial genome reference NC_012920.1. Heteroplasmic mtDNA variation among samples was coded as residuals from a linear regression of the number of reads supporting the alternative allele with site-specific read depth, whole-genome sequencing coverage and sequencing batch included as covariates. Significance thresholds were determined with a Bonferroni correction for multiple testing on a p value threshold of 0.05.

Long-Range PCR on mtDNA and High-Coverage Sequencing of PCR Product

For long-range PCR on 72 human DNA samples from CONVERGE, we designed a pair of primers on D-loop sequences on the mtDNA that did not show sequence similarity with nuclear DNA (forward primer: 5′-TGAGGCCAAATATCATTCTGAGGGGC-3′; reverse primer: 5′-TTTCATCATGCGGAGATGTTGGATGG-3′) for the mtDNA. Long-range PCR using these primers covered the whole of the 16,569 bp of the mitochondrial genome. We performed the PCR on 72 samples with thermal cycling conditions as follows: one 1-min cycle at 98°C, 30 cycles of 10 s at 98°C, and then 8 min 15 s at 72°C, one 10-min cycle at 72°C, and then storing PCR products at 4°C.

For long-range PCR on 12 mouse DNA samples, we designed a pair of primers on D-loop sequences on the mtDNA that did not show sequence similarity with nuclear DNA (forward primer: 5′- CCCAGCTACTACCATCATTCAAGT-3′; reverse primer: 5′- GAGAGATTTTATGGGTGTAATGCGG-3′) for the mtDNA. Long-range PCR using these primers covered the whole of the 16,229 bp of the mitochondrial genome. We performed the PCR on 72 samples with thermal cycling conditions as follows: one 1-min cycle at 98°C, 30 cycles of 10 s at 98°C, 30 s at 68°C, and then 8 min 15 s at 72°C, one 10-min cycle at 72°C, and then storing PCR products at 4°C.

PCR mix for both reactions contain 5 μl 5 × GC buffer, 1 μl 10 mM dNTPs, 5 μl Primer cocktail, 0.5 μl 2 U/μl High-Fidelity DNA Polymerase, 100 ng sample DNA.

The PCR products were then sheared to approximately 500-bp fragments and used to construct libraries for sequencing using Illumina Hiseq4000, yielding 100-bp paired-end reads with average per site read depth of 1,757 for human samples and 1,368 for mouse samples. Sequencing products from this process contains only mtDNA.

Calling of Heteroplasmic Variants in Human and Mouse mtDNA from High-Coverage Sequencing of Long-Range PCR Product

Sequencing reads from human samples were mapped to the human mtDNA reference NC_012920.1 using BWA (v.0.5.6) [29] and Samtools (v.0.1.18) [28], while sequencing reads from mice were mapped using the same software to mtDNA reference sequence in mouse reference genome build GRCm38. We obtained total and per allele read depth for sequencing reads that mapped to the human and mouse mitochondria using Samtools mpileup (v.0.1.18) [28]. Filtering criteria also include a base quality threshold of 20 (Phred-scaled) for bases, and mapping quality of 50 (Phred-scaled) and no more than four mismatches to the reference for reads. To filter out potential mismappings due to nucleotide homopolymer runs on the mtDNA, we only interrogated 14,796 out of 16,569 sites in the human mtDNA sequence and 14,717 out of 16,229 site in the mouse mtDNA sequence, masking out 1,773 and 1,512 sites in the mtDNA for being in or beside homopolymer runs of four or more nucleotides. Sequencing coverage at each site was then independently and randomly down-sampled to 500 reads; sites in any sample covered by fewer than 500 reads were discarded from the analysis, and sites with more than a third of the human and mice samples discarded, respectively, were then completely disregarded for further analysis due to lack of evidence. A site was considered heteroplasmic in a sample if there was presence of two or more alleles each supported by more than or equal to 1% of sequencing reads (five reads out of 500 reads). We then calculated the degree of heteroplasmy per sample per heteroplasmic site.

SUPPLEMENTAL INFORMATION

Supplemental Information includes Supplemental Experimental Procedures, one figure, and five tables and can be found with this article online at http://dx.doi.org/10.1016/j.cub.2015.10.065.

AUTHOR CONTRIBUTIONS

Conceptualization, N.C. and J.F.; Methodology, N.C., S.C., T.M., G.J.H., and J.F.; Validation, N.C., J.L., C.L., X.Z., L.L., Y.C., J.H., and Q.L.; Investigation, N.C., Y.L., S.C., T.M., W.C., and J.F.; Writing – Original Draft, N.C. and J.F; Writing – Review & Editing, N.C., R.M. and J.F.; Funding Acquisition, K.S.K and J.F., Resources, G.J.H., K.S.K., and J.F.; Supervision, J.F.

ACKNOWLEDGMENTS

This work was funded by the Wellcome Trust (WT090532/Z/09/Z, WT083573/Z/07/Z, WT089269/Z/09/Z) and by NIH grant MH-100549. All authors are part of the CONVERGE consortium (China, Oxford, and VCU Experimental Research on Genetic Epidemiology) and gratefully acknowledge the support of all partners in hospitals across China. N.C. is supported by the Agency of Science, Technology and Research (A*STAR) Graduate Academy. T.M. is supported by the Long-term Fellowship from the Human Frontier Science Program. We are extremely grateful to all the families who took part in this study, the midwives for their help in recruiting them, and the whole ALSPAC team, which includes interviewers, computer and laboratory technicians, clerical workers, research scientists, volunteers, managers, receptionists, and nurses. The UK Medical Research Council and the Wellcome Trust (Grant ref: 102215/2/13/2) and the University of Bristol provide core support for ALSPAC.

Received: August 6, 2015
Revised: October 7, 2015
Accepted: October 28, 2015
Published: December 10, 2015

REFERENCES

1. Robin, E.D., and Wong, R. (1988). Mitochondrial DNA molecules and virtual number of mitochondria per cell in mammalian cells. J. Cell. Physiol. *136*, 507–513.

2. Clay Montier, L.L., Deng, J.J., and Bai, Y. (2009). Number matters: control of mammalian mitochondrial DNA copy number. J. Genet. Genomics *36*, 125–131.
3. Tang, Y., Schon, E.A., Wilichowski, E., Vazquez-Memije, M.E., Davidson, E., and King, M.P. (2000). Rearrangements of human mitochondrial DNA (mtDNA): new insights into the regulation of mtDNA copy number and gene expression. Mol. Biol. Cell *11*, 1471–1485.
4. Campbell, C.T., Kolesar, J.E., and Kaufman, B.A. (2012). Mitochondrial transcription factor A regulates mitochondrial transcription initiation, DNA packaging, and genome copy number. Biochim. Biophys. Acta *1819*, 921–929.
5. Johnston, I.G., Gaal, B., Neves, R.P., Enver, T., Iborra, F.J., and Jones, N.S. (2012). Mitochondrial variability as a source of extrinsic cellular noise. PLoS Comput. Biol. *8*, e1002416.
6. Cai, N., Chang, S., Li, Y., Li, Q., Hu, J., Liang, J., Song, L., Kretzschmar, W., Gan, X., Nicod, J., et al. (2015). Molecular signatures of major depression. Curr. Biol. *25*, 1146–1156.
7. CONVERGE Consortium (2015). Sparse whole-genome sequencing identifies two loci for major depressive disorder. Nature *523*, 588–591.
8. Speed, D., Hemani, G., Johnson, M.R., and Balding, D.J. (2012). Improved heritability estimation from genome-wide SNPs. Am. J. Hum. Genet. *91*, 1011–1021.
9. Pruim, R.J., Welch, R.P., Sanna, S., Teslovich, T.M., Chines, P.S., Gliedt, T.P., Boehnke, M., Abecasis, G.R., and Willer, C.J. (2010). LocusZoom: regional visualization of genome-wide association scan results. Bioinformatics *26*, 2336–2337.
10. Boyd, A., Golding, J., Macleod, J., Lawlor, D.A., Fraser, A., Henderson, J., Molloy, L., Ness, A., Ring, S., and Davey Smith, G. (2013). Cohort Profile: the 'children of the 90s'–the index offspring of the Avon Longitudinal Study of Parents and Children. Int. J. Epidemiol. *42*, 111–127.
11. Walter, K., Min, J.L., Huang, J., Crooks, L., Memari, Y., McCarthy, S., Perry, J.R., Xu, C., Futema, M., Lawson, D., et al.; UK10K Consortium (2015). The UK10K project identifies rare variants in health and disease. Nature *526*, 82–90.
12. Ding, J., Sidore, C., Butler, T.J., Wing, M.K., Qian, Y., Meirelles, O., Busonero, F., Tsoi, L.C., Maschio, A., Angius, A., et al. (2015). Assessing Mitochondrial DNA Variation and Copy Number in Lymphocytes of ~2,000 Sardinians Using Tailored Sequencing Analysis Tools. PLoS Genet. *11*, e1005306.
13. Hazkani-Covo, E., Zeller, R.M., and Martin, W. (2010). Molecular poltergeists: mitochondrial DNA copies (numts) in sequenced nuclear genomes. PLoS Genet. *6*, e1000834.
14. Marinov, G.K., Wang, Y.E., Chan, D., and Wold, B.J. (2014). Evidence for site-specific occupancy of the mitochondrial genome by nuclear transcription factors. PLoS ONE *9*, e84713.
15. Abecasis, G.R., Auton, A., Brooks, L.D., DePristo, M.A., Durbin, R.M., Handsaker, R.E., Kang, H.M., Marth, G.T., and McVean, G.A.; 1000 Genomes Project Consortium (2012). An integrated map of genetic variation from 1,092 human genomes. Nature *491*, 56–65.
16. Mercer, T.R., Neph, S., Dinger, M.E., Crawford, J., Smith, M.A., Shearwood, A.M., Haugen, E., Bracken, C.P., Rackham, O., Stamatoyannopoulos, J.A., et al. (2011). The human mitochondrial transcriptome. Cell *146*, 645–658.
17. Lee, S.H., Yang, J., Goddard, M.E., Visscher, P.M., and Wray, N.R. (2012). Estimation of pleiotropy between complex diseases using single-nucleotide polymorphism-derived genomic relationships and restricted maximum likelihood. Bioinformatics *28*, 2540–2542.
18. Ekstrand, M.I., Falkenberg, M., Rantanen, A., Park, C.B., Gaspari, M., Hultenby, K., Rustin, P., Gustafsson, C.M., and Larsson, N.G. (2004). Mitochondrial transcription factor A regulates mtDNA copy number in mammals. Hum. Mol. Genet. *13*, 935–944.
19. Meuer, K., Suppanz, I.E., Lingor, P., Planchamp, V., Göricke, B., Fichtner, L., Braus, G.H., Dietz, G.P., Jakobs, S., Bähr, M., and Weishaupt, J.H. (2007). Cyclin-dependent kinase 5 is an upstream regulator of mitochondrial fission during neuronal apoptosis. Cell Death Differ. *14*, 651–661.
20. Rosenberg, S.M. (2001). Evolving responsively: adaptive mutation. Nat. Rev. Genet. *2*, 504–515.
21. Hastings, P.J., Bull, H.J., Klump, J.R., and Rosenberg, S.M. (2000). Adaptive amplification: an inducible chromosomal instability mechanism. Cell *103*, 723–731.
22. Lee, S.S., Lee, R.Y., Fraser, A.G., Kamath, R.S., Ahringer, J., and Ruvkun, G. (2003). A systematic RNAi screen identifies a critical role for mitochondria in C. elegans longevity. Nat. Genet. *33*, 40–48.
23. Ikeuchi, M., Matsusaka, H., Kang, D., Matsushima, S., Ide, T., Kubota, T., Fujiwara, T., Hamasaki, N., Takeshita, A., Sunagawa, K., and Tsutsui, H. (2005). Overexpression of mitochondrial transcription factor a ameliorates mitochondrial deficiencies and cardiac failure after myocardial infarction. Circulation *112*, 683–690.
24. Gauthier, B.R., Wiederkehr, A., Baquié, M., Dai, C., Powers, A.C., Kerr-Conte, J., Pattou, F., MacDonald, R.J., Ferrer, J., and Wollheim, C.B. (2009). PDX1 deficiency causes mitochondrial dysfunction and defective insulin secretion through TFAM suppression. Cell Metab. *10*, 110–118.
25. Hayashi, Y., Yoshida, M., Yamato, M., Ide, T., Wu, Z., Ochi-Shindou, M., Kanki, T., Kang, D., Sunagawa, K., Tsutsui, H., and Nakanishi, H. (2008). Reverse of age-dependent memory impairment and mitochondrial DNA damage in microglia by an overexpression of human mitochondrial transcription factor a in mice. J. Neurosci. *28*, 8624–8634.
26. Piao, Y., Kim, H.G., Oh, M.S., and Pak, Y.K. (2012). Overexpression of TFAM, NRF-1 and myr-AKT protects the MPP(+)-induced mitochondrial dysfunctions in neuronal cells. Biochim. Biophys. Acta *1820*, 577–585.
27. Suarez, J., Hu, Y., Makino, A., Fricovsky, E., Wang, H., and Dillmann, W.H. (2008). Alterations in mitochondrial function and cytosolic calcium induced by hyperglycemia are restored by mitochondrial transcription factor A in cardiomyocytes. Am. J. Physiol. Cell Physiol. *295*, C1561–C1568.
28. Li, H., Handsaker, B., Wysoker, A., Fennell, T., Ruan, J., Homer, N., Marth, G., Abecasis, G., and Durbin, R.; 1000 Genome Project Data Processing Subgroup (2009). The Sequence Alignment/Map format and SAMtools. Bioinformatics *25*, 2078–2079.
29. Li, H., and Durbin, R. (2009). Fast and accurate short read alignment with Burrows-Wheeler transform. Bioinformatics *25*, 1754–1760.
30. Listgarten, J., Lippert, C., Kadie, C.M., Davidson, R.I., Eskin, E., and Heckerman, D. (2012). Improved linear mixed models for genome-wide association studies. Nat. Methods *9*, 525–526.

Current Biology
Supplemental Information

Genetic Control over mtDNA and Its Relationship to Major Depressive Disorder

Na Cai, Yihan Li , Simon Chang, Jieqin Liang, Chongyun Lin, Xiufei Zhang, Lu Liang, Jingchu Hu, Wharton Chan, Kenneth S Kendler, Tomas Malinauskas, Guo-Jen Huang, Qibin Li, Richard Mott, and Jonathan Flint

Figure S1, Related to Figure1: GWAS on mtDNA with MDD as covariate

A

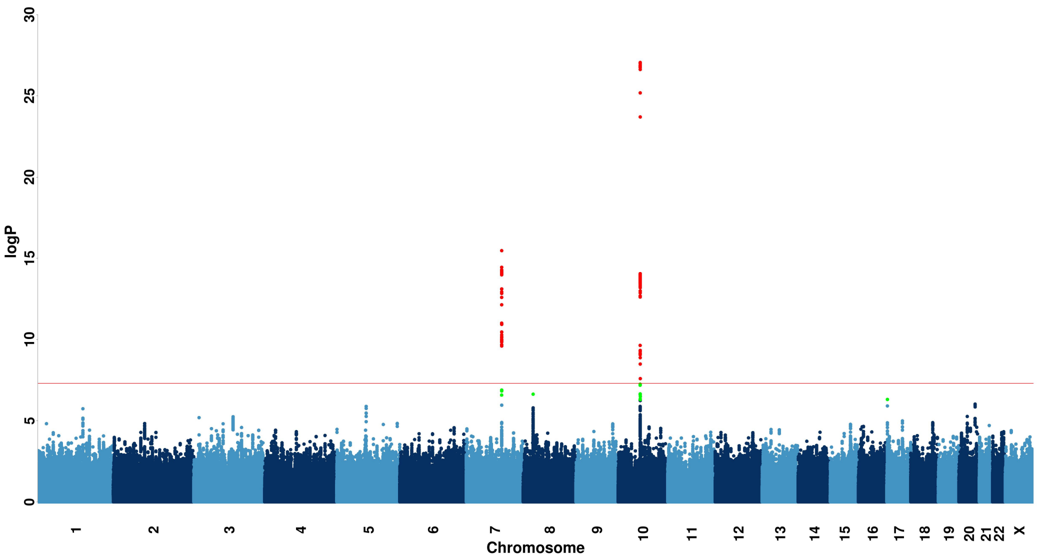

B

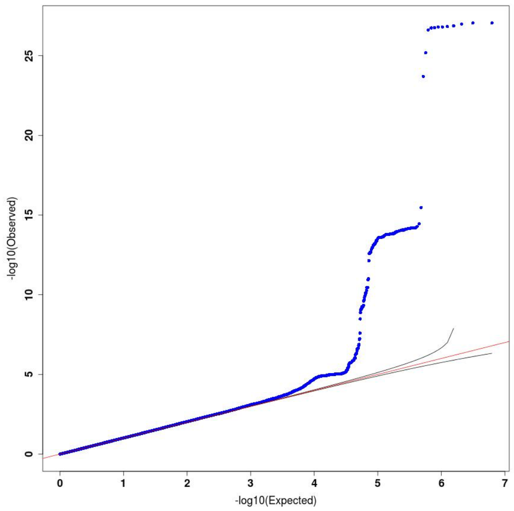

(A) Manhattan plot of genome wide association for amount of mtDNA with MDD as covariate. (B) Quantile-quantile plot of GWAS for amount of mtDNA with MDD as covariate, using leave-one-chromosome-out (LOCO) linear mixed model implemented in FastLMM on 10,442 samples (5,224 cases of MDD, 5,218 controls), genomic control lambda (λ) = 1.017.

Table S1, Related to Table 1: List of SNPs associated with amount of mtDNA with P values < 10^{-6}

CHR	POS	RSID	REF	ALT	FREQ	INFO	HWE_P	P	LOGP
7	92058506	rs10267476	C	T	0.319	0.911	1.20E-06	6.90E-07	6.161
7	92266198	rs147826719	C	T	0.321	0.963	1.20E-01	1.45E-12	11.840
7	92290278	rs41459146	T	C	0.327	0.986	2.40E-01	5.97E-13	12.224
7	92295417	rs2282985	C	G	0.502	0.992	4.90E-01	2.06E-09	8.686
7	92296668	rs56353205	T	C	0.326	0.988	2.30E-01	3.24E-13	12.489
7	92296829	rs4727280	C	T	0.480	0.978	6.60E-01	1.24E-09	8.907
7	92299545	rs2282986	T	C	0.327	0.993	2.80E-01	2.28E-13	12.643
7	92299964	rs2106135	C	T	0.327	0.993	2.80E-01	2.22E-13	12.653
7	92300863	rs2301557	C	T	0.326	0.988	2.50E-01	2.79E-13	12.555
7	92301040	rs9640606	C	T	0.495	0.993	7.10E-01	8.64E-10	9.063
7	92303196	rs10254702	A	G	0.502	0.993	6.70E-01	1.17E-09	8.934
7	92303554	rs3757823	A	G	0.502	0.992	6.80E-01	9.35E-10	9.029
7	92305445	rs75882441	T	A	0.328	0.992	4.20E-01	1.88E-13	12.725
7	92305515	rs6977712	A	T	0.483	0.991	7.10E-01	5.49E-10	9.261
7	92305899	rs6973871	T	C	0.483	0.991	7.40E-01	5.49E-10	9.260
7	92308798	rs12670783	A	G	0.502	0.991	6.70E-01	7.55E-10	9.122
7	92312803	rs2282987	G	C	0.329	0.989	5.80E-01	1.46E-14	13.837
7	92313733	rs2237573	A	G	0.500	0.994	7.10E-01	2.92E-10	9.535
7	92315330	rs6964803	C	T	0.328	0.996	4.20E-01	1.35E-14	13.868
7	92315660	rs11981340	T	C	0.329	0.997	4.90E-01	1.61E-14	13.792
7	92315774	rs11981374	T	C	0.327	0.992	4.60E-01	6.73E-15	14.172
7	92316244	rs13437843	C	T	0.329	0.998	5.00E-01	1.65E-14	13.782
7	92316282	rs60726864	T	A	0.329	0.998	5.00E-01	1.65E-14	13.782
7	92317374	rs2282988	T	C	0.329	0.998	4.90E-01	1.97E-14	13.706
7	92317887	rs10243384	C	T	0.477	0.997	7.70E-01	5.94E-11	10.226
7	92319472	rs11533993	T	C	0.329	0.998	4.90E-01	1.74E-14	13.760
7	92319488	rs11533994	A	C	0.501	0.994	6.30E-01	1.85E-10	9.733
7	92320169	rs10267477	T	C	0.501	0.996	6.00E-01	1.82E-10	9.739
7	92321584	rs1972508	G	A	0.588	0.939	1.90E-02	1.02E-10	9.993
7	92324050	rs11981129	C	T	0.483	0.997	6.00E-01	5.10E-11	10.293
7	92324355	rs10225660	T	C	0.328	0.996	5.10E-01	1.34E-14	13.873
7	92324419	rs17164721	G	T	0.328	0.996	5.40E-01	1.41E-14	13.850
7	92326346	rs2282989	C	T	0.327	0.997	5.30E-01	9.87E-15	14.005
7	92328956	rs1004051	C	A	0.327	0.997	4.70E-01	1.17E-14	13.932
7	92329126	rs3731318	C	T	0.327	0.997	4.70E-01	1.17E-14	13.931
7	92329957	rs1004052	C	G	0.327	0.997	4.90E-01	1.29E-14	13.888
7	92330480	rs3802079	C	T	0.327	0.997	4.70E-01	1.17E-14	13.931
7	92331695	rs2079146	T	C	0.480	0.995	6.70E-01	4.40E-11	10.357
7	92333408	rs10254840	A	G	0.477	0.981	8.80E-01	1.38E-10	9.859
7	92408370	rs445	C	T	0.342	0.906	1.70E-03	6.03E-16	15.219
7	92428239	rs2299242	C	G	0.408	0.923	3.90E-03	5.53E-07	6.257
7	92464778	rs78065312	C	G	0.228	0.903	4.20E-06	3.16E-10	9.500

7	92484093	rs10953073	A	G	0.336	0.910	4.70E-06	3.18E-07	6.497
7	92489813	rs2023699	A	G	0.336	0.910	5.30E-06	3.19E-07	6.496
8	15607313	rs2543154	G	A	0.511	0.984	6.80E-01	2.52E-07	6.598
10	59827743	rs61851889	G	A	0.145	0.997	6.80E-01	9.21E-07	6.036
10	59829407	rs2221296	C	T	0.145	0.998	7.60E-01	8.78E-07	6.057
10	59830413	rs61851890	C	G	0.145	0.998	7.90E-01	9.22E-07	6.035
10	59837321	rs61851911	G	A	0.145	0.999	7.90E-01	9.42E-07	6.026
10	59839893	rs61851913	G	C	0.145	0.998	7.90E-01	9.42E-07	6.026
10	59844227	rs80306558	G	T	0.160	0.991	8.30E-01	3.84E-08	7.416
10	59849616	rs16911771	C	G	0.160	0.988	7.50E-01	4.67E-08	7.331
10	59854651	rs1698469	A	C	0.808	0.987	5.90E-02	7.60E-07	6.119
10	59855663	rs61850576	A	T	0.162	0.987	8.10E-01	4.53E-08	7.344
10	59862105	rs61850577	G	T	0.167	0.979	7.30E-01	2.01E-10	9.697
10	59870890	rs61850578	C	T	0.145	0.992	8.50E-01	5.93E-10	9.227
10	59874268	rs16911810	T	A	0.145	0.992	8.20E-01	5.93E-10	9.227
10	59876328	rs77599563	G	T	0.145	0.992	7.90E-01	5.77E-10	9.239
10	59880706	rs61850584	G	T	0.145	0.989	7.60E-01	4.80E-10	9.319
10	59889478	rs138825781	T	C	0.145	0.992	7.60E-01	4.86E-10	9.313
10	59891501	rs12569763	C	A	0.145	0.991	7.90E-01	4.86E-10	9.314
10	59904889	rs73287122	A	G	0.149	0.994	9.10E-01	1.28E-13	12.893
10	59905514	rs61850589	G	T	0.149	0.994	9.10E-01	1.28E-13	12.893
10	59905849	rs10509086	C	G	0.150	0.995	9.70E-01	1.42E-13	12.846
10	59906486	rs73287129	G	A	0.150	0.995	1.00E+00	1.42E-13	12.846
10	59908601	rs61850590	A	G	0.150	0.993	1.00E+00	1.25E-13	12.904
10	59914787	rs12571364	G	A	0.151	0.993	1.00E+00	3.14E-14	13.503
10	59925048	rs7072206	C	T	0.151	0.992	4.90E-01	2.07E-14	13.683
10	59927787	rs57851212	A	T	0.152	0.993	4.90E-01	1.71E-14	13.766
10	59929700	rs77599079	T	C	0.152	0.992	5.90E-01	5.82E-15	14.235
10	59938336	rs12570088	A	G	0.155	0.994	5.00E-01	6.15E-15	14.211
10	59941791	rs10509088	C	T	0.156	0.999	9.40E-01	6.40E-15	14.194
10	59946672	rs1199105	T	C	0.156	0.999	9.20E-01	1.25E-14	13.902
10	59947543	rs12252749	A	T	0.156	0.999	9.40E-01	1.13E-14	13.948
10	59957000	rs77350161	G	A	0.156	0.995	9.40E-01	1.09E-14	13.963
10	59957071	rs12252003	A	C	0.149	0.938	2.10E-01	7.73E-15	14.112
10	59960083	rs1625716	T	G	0.155	0.998	8.90E-01	1.11E-14	13.957
10	59966533	rs58375950	C	T	0.155	0.998	8.30E-01	1.21E-14	13.918
10	59967203	rs60679872	C	T	0.155	0.999	8.60E-01	1.21E-14	13.918
10	59969111	rs12261547	G	C	0.155	0.999	8.30E-01	1.21E-14	13.918
10	59970430	rs61875332	G	T	0.155	0.999	8.60E-01	1.21E-14	13.918
10	59971818	rs12251970	C	T	0.155	0.998	8.60E-01	1.21E-14	13.917
10	59974730	rs61875333	G	C	0.155	0.998	8.00E-01	1.21E-14	13.916
10	59987662	rs12572236	G	T	0.155	0.998	8.60E-01	8.60E-15	14.065
10	59990325	rs11006087	C	T	0.155	0.998	8.00E-01	8.60E-15	14.065
10	60001675	rs7900114	C	T	0.155	0.998	8.00E-01	1.01E-14	13.994
10	60004329	rs139600710	G	C	0.155	0.995	6.20E-01	8.62E-15	14.064
10	60023959	rs61873887	G	A	0.155	0.998	8.60E-01	8.33E-15	14.079

10	60031460	rs12572520	T	G	0.155	0.998	7.80E-01	8.22E-15	14.085
10	60032636	rs2790176	A	G	0.155	0.998	7.80E-01	8.22E-15	14.085
10	60035152	rs2275442	C	T	0.155	0.998	7.80E-01	8.22E-15	14.085
10	60047177	rs3758567	G	C	0.155	0.999	8.00E-01	1.21E-14	13.917
10	60049790	rs12241783	G	T	0.155	0.999	7.50E-01	1.01E-14	13.997
10	60052201	rs11006105	C	T	0.155	0.999	7.50E-01	1.01E-14	13.997
10	60052999	rs12264744	C	T	0.155	0.999	7.50E-01	1.01E-14	13.997
10	60065855	rs59030640	G	A	0.154	0.990	8.60E-01	7.57E-15	14.121
10	60084394	rs112187169	C	A	0.151	0.985	4.80E-01	9.81E-14	13.008
10	60092695	rs61873953	G	A	0.156	0.994	2.40E-01	8.43E-15	14.074
10	60095105	rs112660736	G	T	0.137	0.958	5.00E-01	9.84E-15	14.007
10	60095266	rs16912153	G	A	0.137	0.958	5.00E-01	9.86E-15	14.006
10	60099333	rs59474782	G	A	0.146	0.942	6.00E-03	9.23E-14	13.035
10	60100089	rs4948291	C	T	0.158	0.994	4.80E-01	1.83E-14	13.738
10	60106835	rs1963927	G	T	0.462	0.996	5.00E-01	1.27E-07	6.895
10	60116030	rs12255735	G	C	0.156	0.993	2.30E-01	7.47E-15	14.127
10	60118681	rs10826172	T	C	0.462	0.996	5.40E-01	1.13E-07	6.947
10	60121976	rs11006120	C	G	0.158	0.993	4.60E-01	1.19E-14	13.926
10	60122066	rs11006121	C	T	0.463	0.991	3.30E-01	1.14E-07	6.943
10	60122678	rs4145785	A	T	0.462	0.996	5.00E-01	1.16E-07	6.937
10	60125877	rs11006122	C	T	0.462	0.996	4.80E-01	1.14E-07	6.942
10	60127410	rs10826174	G	C	0.464	0.989	4.40E-01	1.06E-07	6.974
10	60142116	rs9971282	C	T	0.162	0.956	6.40E-02	2.39E-27	26.622
10	60142402	rs11006125	A	T	0.168	0.982	8.30E-02	1.11E-27	26.957
10	60142800	rs9971104	G	T	0.168	0.984	9.00E-02	9.16E-28	27.038
10	60142880	rs11006126	T	C	0.169	0.978	9.00E-02	8.73E-28	27.059
10	60144207	rs4390300	G	A	0.489	0.987	1.40E-03	7.22E-07	6.141
10	60144884	rs2279340	C	T	0.171	0.990	3.50E-01	2.76E-27	26.558
10	60144998	rs2279339	C	A	0.170	0.992	3.50E-01	2.74E-27	26.563
10	60145079	rs12247015	A	G	0.173	0.979	3.90E-01	1.99E-27	26.701
10	60145342	rs1937	G	C	0.170	0.989	4.10E-01	2.47E-27	26.608
10	60145597	rs4397793	G	C	0.494	0.984	2.70E-03	7.02E-07	6.154
10	60147270	rs10826178	G	A	0.491	0.997	2.60E-03	6.80E-07	6.168
10	60147784	rs10763537	G	A	0.491	0.997	3.00E-03	6.24E-07	6.205
10	60148692	rs2306604	A	G	0.492	0.993	2.50E-03	5.79E-07	6.238
10	60151026	rs10826179	A	G	0.492	0.996	1.90E-03	6.00E-07	6.222
10	60155120	rs1049432	G	T	0.172	0.995	4.90E-01	2.95E-27	26.530
10	60156166	rs11006132	A	G	0.172	0.995	5.10E-01	2.83E-27	26.548
10	60156584	rs11006133	G	A	0.491	0.993	2.30E-03	5.68E-07	6.246
10	60157339	rs7089361	C	T	0.491	0.992	2.10E-03	5.47E-07	6.262
10	60158385	rs12259591	G	A	0.489	0.997	1.60E-03	6.11E-07	6.214
10	60158446	rs12254586	T	C	0.489	0.997	1.80E-03	6.12E-07	6.213
10	60159362	rs12245545	T	G	0.489	0.996	1.60E-03	6.15E-07	6.211
10	60160312	rs7077225	G	T	0.490	0.991	2.60E-03	6.42E-07	6.192
10	60160455	rs16912212	A	G	0.489	0.996	1.30E-03	6.04E-07	6.219
10	60161172	rs4525176	G	A	0.491	0.994	1.00E-03	4.97E-07	6.304

10	60161640	rs10763540	A	G	0.491	0.994	1.20E-03	4.97E-07	6.304
10	60164560	rs11006137	G	A	0.499	0.984	1.10E-03	1.54E-07	6.813
10	60164820	rs10826182	C	T	0.499	0.984	1.10E-03	1.59E-07	6.798
10	60167882	rs6481387	C	T	0.499	0.984	2.30E-03	1.45E-07	6.840
10	60168003	rs7905675	G	A	0.499	0.984	2.30E-03	1.45E-07	6.840
10	60169880	rs11817790	G	T	0.499	0.983	2.50E-03	1.48E-07	6.830
10	60175440	rs61190999	C	T	0.172	0.962	4.50E-01	2.96E-25	24.529
10	60193608	rs76455223	T	A	0.171	0.933	9.40E-01	1.38E-23	22.861
10	60228964	rs61875518	G	A	0.157	0.967	1.20E-01	1.12E-13	12.952
10	60229347	rs1427215	G	A	0.157	0.970	5.60E-02	1.37E-13	12.862
10	60230671	rs61875520	C	G	0.160	0.989	3.80E-01	1.51E-13	12.820
10	60231657	rs7093118	T	C	0.160	0.989	3.80E-01	1.51E-13	12.820
10	60240354	rs17644676	A	G	0.161	0.990	4.80E-01	2.15E-13	12.669
10	60241036	rs35258735	T	A	0.161	0.990	4.80E-01	2.15E-13	12.669
10	60244111	rs61875523	A	G	0.161	0.990	4.80E-01	2.16E-13	12.666
10	60245124	rs77192138	G	A	0.156	0.983	5.90E-01	4.60E-13	12.337
10	60247792	rs61875524	C	T	0.124	0.997	2.30E-01	2.03E-09	8.693
10	60257846	rs74335736	G	A	0.124	0.998	2.90E-01	2.28E-09	8.642
10	60258420	rs77095691	T	C	0.124	0.998	2.90E-01	2.28E-09	8.642
10	60261176	rs76383270	C	T	0.124	0.998	2.90E-01	2.28E-09	8.642
10	60261367	rs76110033	A	T	0.124	0.998	2.90E-01	2.28E-09	8.642
10	60262181	rs78111290	T	C	0.124	0.998	2.90E-01	2.28E-09	8.642
10	60264332	rs1427208	T	A	0.124	0.991	3.60E-01	4.94E-09	8.307
10	60264609	rs11006167	G	A	0.127	0.972	3.40E-01	8.44E-09	8.073
10	60293094	rs12571046	A	C	0.107	0.936	4.90E-01	4.91E-08	7.309
10	60374087	rs11006205	A	C	0.107	0.945	6.30E-01	7.99E-07	6.097

This table shows the list of SNPs associated with amount of mtDNA quantified from 1.7X coverage next generation sequencing on 10,442 samples with P values < 10^{-6} in a leave-one-chromosome-out linear-mixed model implemented in FastLMM. The first five columns contain the chromosome (CHR), position (POS), SNP identifier (RSID), and reference (REF) and alternative (ALT) alleles; the next three columns show the alternative allele frequency (FREQ) of the SNPs in CONVERGE, the IMPUTE2-style imputation information score (IMPUTE2-INFO) of the imputed allele dosages, and the P-value of violation of Hardy Weinberg Equilibrium (HWE_P); the last two columns show the P-value of association of the SNPs with amount of mtDNA in CONVERGE in a linear mixed-model (P) and the –log10 of the P-value (LOGP).

Table S2, Related to Experimental Procedures: Frequency of diagnostic alleles for mitochondrial haplogroups

Haplogroup	Diagnostic site	Reference Allele	Diagnostic Allele	Frequency (%)					
				Central Asia	East Asia	Han Chinese	1000G	1000G CHSCHB	CONVERGE
A	663	A	G	7.00	7.00	4.00-16.70	5.54	6.02	7.21
B	8280-8289	ACCCCCTCTA	A	5.00	16.00	0.60-3.30	NA	NA	NA
C	13263	A	G	12.00	5.00	2.00-8.00	3.04	3.24	3.98
D	5178	C	A	15.00	26.00	1.40-3.30	5.15	22.55	21.67
E	13626	C	T	0.00	0.00	NA	NA	NA	0.09
F	6392	T	C	5.00	11.00	1.40-6.00	3.71	14.42	14.34
G	4833	A	G	5.00	4.00	NA	0.00	0.00	14.77
H	7028	C	C	15.00	1.00	NA	11.37	0.00	NA
I	4529	A	T	1.00	0.00	NA	0.05	0.00	NA
J	13708	G	A	3.00	1.00	NA	5.27	5.56	4.31
K	12308	A	G	1.00	0.00	NA	NA	NA	NA
L	3594	C	T	0.00	0.00	NA	15.78	0.00	0.02
S	8404	T	C	0.00	0.00	NA	0.19	0.92	0.28
T	4917	A	G	6.00	0.00	NA	0.22	0.00	0.28
U	12308	A	G	10.00	0.00	NA	NA	NA	NA
V	4580	G	A	0.00	0.00	NA	NA	NA	NA
W	11947	A	G	2.00	0.00	NA	0.96	0.00	0.06
X	6371	C	T	0.00	0.00	NA	0.43	0.00	0.02
Y	8392	G	A	1.00	1.00	1.30-3.80	0.19	0.00	NA
Z	9090	T	C	2.00	2.00	1.30-7.10	0.31	1.85	3.24

This table shows in the first four columns the mitochondrial haplogroup, diagnostic diagnostic site for the haplogroup, the reference allele in the human mtDNA reference NC_012920.1, and the diagnostic allele. The next six columns show the frequency (%) of occurrence of the diagnostic allele in Central Asians and East Asians from MitoMap[S1], estimates from multiple studies in Han Chinese population[S2], all 1000 Genomes Phase 3 samples, 1000 Genomes Phase 3 Chinese Beijing (CHB) and Chinese South (CHS) samples, and CONVERGE study samples.

Table S3, Related to Experimental Procedures: Correlation between PC1 in CONVERGE and four homoplasmic variants adaptive to arctic climate

Variant position	Adaptive allele	Haplogroup	Gene	Amino acid change	Linear regression P value with PC1	Coefficient	Allele frequency
4824	G	A	ND2	T112A	NA	NA	NA
8794	T	A	ATP6	H90Y	NA	NA	NA
11969	A	C	ND4	A404T	0.00056	0.0022	0.020
15204	C	C	cytb	I153T	0.00290	0.0025	0.013

This table shows in the first three columns position, adaptive allele, haplogroup in which four previously identified homoplasmic variants reported to be present in Asian mitochondrial Haplogroups (A, C, D and X) [S3] and associated with colder climates. The next two columns show the genes which the variants reside in, and the amino acid changes they cause. The next two columns show the association P value and regression coefficient between the presence of the adaptive alleles and PC1 in CONVERGE (Experimental Procedures), which captures the North-South cline. The final column shows the allele frequency of the adaptive alleles in CONVERGE. Two sites at positions 4824 and 8794 were not called in CONVERGE due to lack of coverage.

Table S4, Related to Experimental Procedures: Correlation between degree of heteroplasmy called in 72 samples from low coverage sequencing and high-coverage sequencing of long range PCR of mtDNA

Position	Mean degree of heteroplasmy		Pearson r2	P-value
	Low coverage	Long range PCR		
**MT146**	**0.118**	**0.126**	**0.999**	**0.000**
**MT451**	**0.011**	**0.012**	**0.999**	**0.000**
MT512	0.000	0.006	NA	NA
**MT513**	**0.374**	**0.411**	**0.984**	**0.000**
MT567	0.004	0.011	0.999	0.000
MT955	0.000	0.002	NA	NA
MT1290	0.000	0.000	NA	NA
MT2226	0.000	0.000	NA	NA
MT2487	0.010	0.000	0.013	0.340
MT3167	0.000	0.000	NA	NA
MT3243	0.000	0.000	NA	NA
MT3447	0.000	0.001	NA	NA
**MT5894**	**0.033**	**0.060**	**0.829**	**0.000**
MT6289	0.000	0.001	NA	NA
MT10283	0.000	0.000	0.003	0.663
MT10306	0.027	0.000	0.000	0.942
MT11031	0.001	0.005	0.000	0.911
MT11866	0.000	0.002	NA	NA
MT12417	0.002	0.005	0.013	0.341
MT12684	0.000	0.000	0.004	0.615
MT15536	0.000	0.000	NA	NA
MT15900	0.000	0.000	0.001	0.806
**MT15939**	**0.012**	**0.012**	**1.000**	**0.000**
**MT16129**	**0.183**	**0.183**	**0.993**	**0.000**
MT16179	0.003	0.057	0.003	0.657
MT16496	0.002	0.092	0.017	0.285

This table shows the degrees of heteroplasmy in 72 samples for whom there were both low-coverage sequencing data and high coverage sequencing data from long range PCR of mtDNA. The first column shows the 26 sites in the mtDNA identified to be heteroplasmic at an occurrence of higher than 0.1% in CONVERGE. The next two columns show the mean degree of heteroplasmy among the 72 samples at each of the 26 sites, quantified from low coverage sequencing and high coverage sequencing of long range PCR product on mtDNA respectively. Where the mean degree of heteroplasmy is 0 in either columns, there was no heteroplasmy detected in any of the 72 samples using the relevant dataset. The next two columns show the per site Pearson correlation r2 and

between the degree of heteroplasmy called from low coverage sequencing and high coverage sequencing of long range PCR on mtDNA in 72 sample, and the P-value of the correlation. Six sites highlighted in bold showed mean degrees of heteroplasmy of over 1% in both datasets and were highly correlated, and were included in association testing between site-specific degree of heteroplasmy and amount of mtDNA.

Table S5, Related to Table 3: Association between degree of heteroplasmy at six heteroplasmic sites with MDD

MARKER	REF	ALT	FREQ	TYPE	GENE	MDD			
						EFFECT	VAREXP	P	LOGP
MT146	T	C	0.005	upstream	RNR1,tRNA-Phe	1.275	0.003	2.39E-01	0.622
MT451	A	I	0.002	regulatory	NA	0.734	0.000	8.39E-02	1.076
MT513	G	I	0.416	regulatory	NA	-0.035	0.000	2.31E-01	0.636
MT5894	A	I	0.017	regulatory	NA	-0.582	0.001	3.15E-01	0.502
MT15939	C	I	0.014	regulatory	NA	0.051	0.000	1.66E-01	0.780
MT16129	G	A	0.020	upstream;downstream	tRNA-Pro;CYTB,tRNA-Thr	-0.229	0.000	2.16E-01	0.665

This table shows the association between degree of heteroplasmy at four heteroplasmic sites in the mtDNA and major depressive disorder (MDD) in 10,442 samples. The first four columns show the position of the heteroplasmic site in mtDNA (MARKER), reference allele in the human mitochondrial genome reference NC_012920.1 (REF), the alternative allele (ALT) for which the heteroplasmy is detected, and the frequency of occurrence of this heteroplasmy in the cohort (FREQ). An "I" in the ALT column means the heteroplasmy is for an insertion or deletion mutation. The next two columns show characterisitics of the four heteroplasmic sites: annotation of variant function (ANNOTATION) and nearest gene (GENE). The final four columns show the results of association testing between degree of heteroplasmy at each site with amount of mtDNA by logistic regression: direction of effect (EFFECT) from linear regression, variance of amount of mtDNA explained by the site heteroplasmy (VAREXP) from difference in residual sum of squares in analysis of variance (ANOVA) between the model with and without degree of heteroplasmy as the test term in logistic regression, P value of association (P) between degree of heteroplasmy and MDD, and –log10 of the P value (LOGP). Degree of heteroplasmy at none of the sites were significantly associated with MDD.

Supplemental Experimental Procedures

1. Verifying homoplasmic mtDNA variation calls

As accurate calls of mtDNA homoplasmic variants form the basis of association analyses and are important in preventing misidentification of heteroplasmic variants in the mtDNA, we checked for both sensitivity and accuracy in calling homoplasmic variants from low-coverage whole-genome sequencing data. The strategy to call homoplasmic variants was to look for non-reference alleles supported by more than 90% of the reads at any particular site in each sample, but only at those sites where there are more than 10 uniquely mapped reads of high mapping quality (>59 in Phred scale).

Sensitivity of variant calling

To estimate the sensitivity of our variant calling method, we applied our method of homoplasmic variant calling on the 1000 Genomes Phase 3 whole-genome sequencing data (n=2,598, of which 216 are from Han Chinese populations, CHB and CHS). We compared the set of common homoplasmic variants (>0.1%) we called in CONVERGE with homoplasmic variants called in 1000 Genomes Project Phase 3 samples. 948 out of 1,030 (88.85%) of the common homoplasmic variants we found in CONVERGE were found to be polymorphic in 1000 Genomes Phase 3, and 546 of them (51.17%) were found to be occurring at >0.1% frequency in the Han Chinese populations.

Presence of mtDNA haplogroup markers

We checked the allele frequencies of homoplasmic variant sites called in CONVERGE at diagnostic SNPs for haplogroups against previously documented frequencies in Central Asians and East Asians in MitoMap [S1], the combined estimates of allele frequencies from previous studies on Han Chinese populations [S2], as well as allele frequencies called from all 1000 Genomes Phase 3 samples and those from Han Chinese populations (Table S2). The frequencies of alleles at SNPs diagnostic for the different haplogroups are highly consistent with those previously reported.

We checked for the presence of four homoplasmic variants previously reported to be present in Asian mitochondrial Haplogroups (A, C, D and X) [S3] and associated with colder climates in our sample. Two of the four variants were identified as homoplasmic

variants occurring at frequencies above 0.1% in our sample, and were found to be associated with PC1, capturing the North-South cline of geographical origin of our samples, calculated from eigen decomposition of the a genetic relatedness matrix calculated from 322,911 common SNPs of minor allele frequency (MAF) > 1% and linkage disequilibrium $r^2 < 0.5$ using GCTA (version 1.24.4, Methods)[S4] (Table S3). Two variants were not called in our dataset due to lack of coverage at the sites.

2. Verifying heteroplasmic mtDNA variation calls by long range polymerase chain reaction (PCR) and sequencing of mitochondrial DNA

To identify and quantify the number of heteroplasmic sites independent of sequence coverage we down-sampled the mtDNA reads so that each site was covered by 50 reads. We disregarded sites at which more than 10% of individuals did not fulfill this criterion, so that estimates of heteroplasmy from all remaining sites were based on equal coverage on an adequate sample size. We required the presence of two alleles, each supported by two or more reads at sites and occurring at higher than 0.1% frequency in the cohort (10 individuals), for a site to be considered heteroplasmic. Using these criteria we identified heteroplasmy at 26 positions in the mtDNA.

Low-coverage sequencing has a high false-negative rate in the calling of heteroplasmic sites, and some of the heteroplasmies we detected using low-coverage sequencing might be reads originating from NUMTs mapping incorrectly to the mtDNA reference. Therefore we performed the same analysis using the high-coverage sequencing on 72 samples we obtained with long-range PCR, ensuring both high-coverage and mtDNA origin of sequencing reads.

We quantified heteroplasmy in the 72 samples, after down-sampling the high-coverage sequencing to 500 reads per site and discarding those samples with fewer than 500 reads covering at each site. We only counted heteroplasmic sites where the fraction of reads supporting the minor heteroplasmic allele was higher than 1% (supported by a minimum of 5 reads). This yielded 408 such sites. Comparison between mean degree of heteroplasmy obtained from low-coverage sequencing on 72 samples and high-coverage sequencing of products of long range PCR on mtDNA on the same samples showed that when the mean degree of heteroplasmy among samples is high (10% or higher) in both low-coverage sequencing and long range PCR data, the correlation between the two estimates is high (Table S4). For lower degrees of heteroplasmy, this correlation drops substantially. Therefore we considered further only sites where heteroplasmy rates are greater than 10% in an individual.

Supplemental References

S1. Lott, M.T., et al., *mtDNA Variation and Analysis Using MITOMAP and MITOMASTER.* Curr Protoc Bioinformatics, 2013. **1**(123): p. 1 23 1-1 23 26.

S2. Yao, Y.G., et al., *Phylogeographic differentiation of mitochondrial DNA in Han Chinese.* Am J Hum Genet, 2002. **70**(3): p. 635-51.

S3. Ruiz-Pesini, E., et al., *Effects of purifying and adaptive selection on regional variation in human mtDNA.* Science, 2004. **303**(5655): p. 223-6.

S4. Yang, J., et al., *GCTA: a tool for genome-wide complex trait analysis.* Am J Hum Genet, 2011. **88**(1): p. 76-82.
